# Comparative DNA methylation among females with neurodevelopmental disorders and seizures identifies *TAC1* as a MeCP2 target gene

**DOI:** 10.1186/1866-1955-5-15

**Published:** 2013-06-11

**Authors:** Kimberly A Aldinger, Jasmine T Plummer, Pat Levitt

**Affiliations:** 1 Zilkha Neurogenetic Institute, Keck School of Medicine of USC, 1501 San Pablo Street, Los Angeles, CA 90089, USA; 2 Department of Cell & Neurobiology, Keck School of Medicine of USC, Los Angeles, CA 90089, USA

**Keywords:** DNA methylation, MeCP2, Epigenetics, Autism, Rett syndrome, Epilepsy

## Abstract

**Background:**

Several proteins involved in epigenetic regulation cause syndromic neurodevelopmental disorders when human genes are mutated. More general involvement of epigenetic mechanisms in neurodevelopmental phenotypes is unclear.

**Methods:**

In an attempt to determine whether DNA methylation differentiates clinical subgroups, profiling was performed on bisulfite converted DNA from lymphoblastoid cell lines (LCLs) in discovery (*n* = 20) and replication (*n* = 40) cohorts of females with Rett syndrome (RTT; *n* = 18), autism (AUT; *n* = 17), seizure disorder (SEZ; *n* = 6), and controls (CTL; *n* = 19) using Illumina HumanMethylation27 arrays. *TAC1* CpGs were validated using a Sequenom EpiTYPER assay and expression was measured in LCLs and postmortem brain. Chromatin immunoprecipitation was performed in HEK cells. Cells were treated with valproic acid and MeCP2 binding was assessed.

**Results:**

Two female-only cohorts were analyzed. DNA methylation profiling in a discovery cohort identified 40 CpGs that exhibited statistically significant differential methylation (≥15%) between clinical groups (*P* <0.01). Hierarchical clustering and principal components analysis suggested neurodevelopmental groups were distinct from CTL, but not from each other. In a larger and more heterogeneous replication cohort, these 40 CpG sites suggested no clear difference between clinical groups. Pooled analysis of DNA methylation across all 60 samples suggested only four differentially methylated CpG sites (*P* <0.0005), including *TAC1*. *TAC1* promoter CpG hypermethylation was validated in AUT and SEZ (*P* <0.005). Analyzed for the first time in postmortem brain, *TAC1* expression was reduced in cingulate cortex in RTT and AUT+SEZ (*P* = 0.003). However, no significant difference in *TAC1* promoter CpG methylation was detected in RTT and AUT+SEZ brains. Additional molecular analyses revealed that MeCP2 binds directly to the *TAC1* promoter and is sensitive to antiepileptic drug treatment.

**Conclusion:**

These data suggest that DNA methylation is not widely altered in RTT, consistent with subtle changes in gene expression previously observed. However, *TAC1* may be an important target for further functional analyses in RTT. Studies of larger sample cohorts using primary cells that also consider shared clinical features and drug treatments may be required to address apparent subtle disruptions of DNA methylation in neurodevelopmental disorders.

## Background

DNA methylation is an epigenetic modification essential for a range of cellular functions including local and global transcription, genomic integrity, X chromosome inactivation, and genomic imprinting [1]. Epigenetic mechanisms are critical for a variety of neurobiological and cognitive processes including neurogenesis, stem cell maintenance [2], synaptic plasticity [3], learning and memory [4], and social imprinting [5]. The importance of epigenetic processes in normal brain function and development is further illustrated by the presence of neurodevelopmental deficits in syndromic disorders caused by mutations in genes associated with these processes. Rett syndrome (RTT), caused by mutations in the gene encoding methyl CpG binding protein 2 (*MECP2*), which binds to methylated DNA, is one such example [6].

DNA methylation is a covalent modification of the cytosine nucleotide that occurs in vertebrates at CpG dinucleotides to silence gene transcription directly, by inhibiting transcription factor binding, and indirectly, by recruiting methyl-CpG-binding proteins that engage in chromatin remodeling activities [7]. CpG islands located proximal to gene promoters contain a high density of CpG sites, yet they are often hypomethylated [8]. Nonetheless, differential DNA methylation at CpG islands, first identified in cancer, has more recently been associated with various neurological and neuropsychiatric disorders [1,9,10].

We investigated whether lymphoblastoid cell line (LCL) DNA methylation profiles could be used to differentiate related neurodevelopmental disorders into clinical categories and identify specific target genes that might be relevant to pathophysiology. We also examined whether differential DNA methylation identified at gene promoters in a transformed peripheral tissue among distinct clinical groups reflects changes in gene expression in postmortem brain tissue. Finally, because seizures often co-occur in the neurodevelopmental disorders examined here, we also investigated the effects of valproic acid (VPA), a potent antiepileptic drug (AED) and histone deacetylase inhibitor, on MeCP2 binding.

## Methods

### Samples

Genomic DNA from females with RTT or seizure disorder (SEZ) was obtained from the Coriell Cell Repository [11]. Genomic DNA from age-matched females with autism (AUT) or controls (CTLs) was selected from the NIMH Center for Collaborative Genetic Studies on Mental Disorders Autism Pedigrees v5.0 [12]. All 60 DNA samples (Additional file 1: Table S1) used for methylation assays originated from LCLs. Fresh-frozen postmortem brain samples were obtained through the Autism Speaks-supported Autism Tissue Program at the Harvard Brain Tissue Resource Center [13] or the Eunice Kennedy Shriver National Institute of Child Health and Human Development Brain and Tissue Bank at the University of Maryland School of Medicine [14]. Tissue samples of the striatum, cingulate and temporal cortices were obtained from five RTT females and five CTL females (Additional file 1: Table S3). Additional samples of cingulate and temporal cortices were obtained from five AUT females and three additional CTL females.

### Genome-wide promoter CpG DNA methylation profiling

Genomic DNA was bisulfite modified using the EZ DNA Methylation Kit (Zymo Research, Orange, CA, USA) and hybridized to the Illumina (San Diego, CA, USA) Infinium HumanMethylation27 BeadChip by the USC Epigenome Center as described elsewhere [15,16]. To evaluate array fidelity, DNA aliquots for six samples per clinical group from the discovery cohort were included in the replication cohort. The β values (0 to 1.0, where 0 represents no methylation and 1 represents complete methylation) for each CpG site were calculated as the methylated signal intensity divided by the sum of the methylated and unmethylated signals. Loci that possibly contained SNPs and/or repetitive elements were removed, leaving 21,583 probes representing CpG sites in 12,936 genes for analysis. Two AUT samples were excluded due to sex discrepancy (Additional file 1: Figure S1). No additional major chromosomal abnormalities were apparent. Copy number variants were previously reported for 11 samples included in this study (Additional file 1: Table S4).

### TAC1 DNA methylation validation

The validation experiment included 56 samples from the Illumina discovery and replication cohorts. Another LCL DNA aliquot was bisulfite modified using the EZ DNA Methylation Kit (Zymo Research) by the USC Epigenome Center, according to the manufacturer’s protocol. Genomic DNA was isolated from gray matter dissected from the cingulate cortex using the Wizard Genomic DNA Purification Kit (Promega, Madison, WI, USA) and was bisulfite modified. Sequenom primers (Additional file 1: Table S5) were designed to target the region that overlapped with Illumina probe location using the EpiDesigner BETA software [17]. The Sequenom (San Diego, CA, USA) MALDI-TOF mass spectrometry EpiTYPER Assay was performed by the Vanderbilt University Center for Human Genetics Research DNA Resources Core according to the manufacturer’s protocol.

### TAC1 mRNA expression

Total RNA was isolated from LCLs using the Illustra TriplePrep Kit (GE Healthcare Bio-Sciences Corp., Piscataway, NJ, USA) according to the manufacturer’s protocol. Total RNA was isolated from gray matter-dissected brain tissue or HEK cell pellets using the RNeasy Plus Mini Kit (Qiagen, Valencia, CA, USA) according to the manufacturer’s protocol. RNA quality was determined using an Agilent (Palo Alto, CA, USA) Bioanalyzer 2100 system; the RNA integrity number was >8 for all samples. cDNA was synthesized using the SuperScript III First Strand cDNA Synthesis Kit (Invitrogen, Carlsbad, CA, USA) according to the manufacturer’s protocol using oligo dT primers. Quantitative RT-PCR was performed in triplicate for each sample using the Power SYBR Green PCR master mix (Life Technologies Corp., Carlsbad, CA, USA) and target-specific primers (Additional file 1: Table S4) according to the manufacturer’s recommendations. Assays were analyzed using a CFX96 Real-Time PCR detection system (Bio-Rad, Hercules, CA, USA). The delta cycle threshold (ΔCt) of target relative to *ACTB* was averaged per sample.

### MeCP2 chromatin immunoprecipitation

The EZ-Magna ChIP A/G Chromatin Immunoprecipitation Kit (EMD Millipore Corporation, Billerica, MA, USA) was used with slight modifications to the manufacturer’s protocol, as described in Additional file 1.

### Valproic acid treatment

HEK cells and CTL LCLs were grown using standard conditions. After 24 hours, media was replaced with media containing 3 mM VPA (Sigma-Aldrich Corp., St. Louis, MO, USA) and cells were grown for an additional 24 hours. Cells were processed for chromatin immunoprecipitation, as described above, or genomic DNA was extracted using a standard phenol:chloroform protocol and bisulfite modified for the *TAC1* Sequenom EpiTYPER Assay, as described above.

### Statistical analysis

lllumina probes with mean |Δβ| ≥0.15 between pairs of clinical groups within the discovery cohort were first selected for analysis (*n* = 391). Analysis of variance (ANOVA) and the false discovery rate (FDR) were performed to test the association between clinical group and DNA methylation for the selected probes. Illumina probes with *P* <0.01 and FDR <0.10 in the discovery cohort were evaluated in the replication cohort and in a pooled analysis that included all samples. Hierarchical clustering analysis (Ward’s method) using dissimilarity matrices (1 – Pearson’s correlation) and principal component analysis were performed to identify subjects with similar DNA methylation patterns. Statistical analyses were performed using R software [18].

Tukey *post-hoc* analysis was performed to identify pair-wise associations between clinical groups and DNA methylation for the probe that was significantly differentially methylated among clinical groups (cg14224417; *TAC1*). ANOVA and Tukey *post-hoc* tests were performed for each *TAC1* promoter CpG site measured by the Sequenom assay. *t* tests were used to assess the significance of *TAC1* expression (ΔCt) stratified by clinical group for each brain region and to assess the treatment effect in cell lines. To investigate the association between DNA methylation and mRNA expression in the cingulate cortex, stratified by clinical group, we fitted linear regression models. Statistical analyses were performed using SPSS version 18.0.3 software (IBM, Somers, NY, USA).

## Results

### Genome-wide CpG DNA methylation analysis: discovery cohort

Genome-wide DNA methylation profiling using LCLs from females diagnosed with a neurodevelopmental disorder and age-matched controls was performed in two independent subject cohorts: a discovery cohort (*n* = 20) and a replication cohort (*n* = 40). The discovery cohort contained LCL samples from females diagnosed with RTT (*n* = 10), AUT (*n* = 4), or CTL (*n* = 6) (Additional file 1: Table S1). In discovery cohort samples, most probes had low DNA methylation levels (median = 0.04 to 0.06; mean = 0.21 to 0.24; range = 0.01 to 0.99). Correlations among discovery cohort samples were similar across probes (Pearson’s *r* >0.98), consistent with previous reports [19-24].

**Figure 1:**
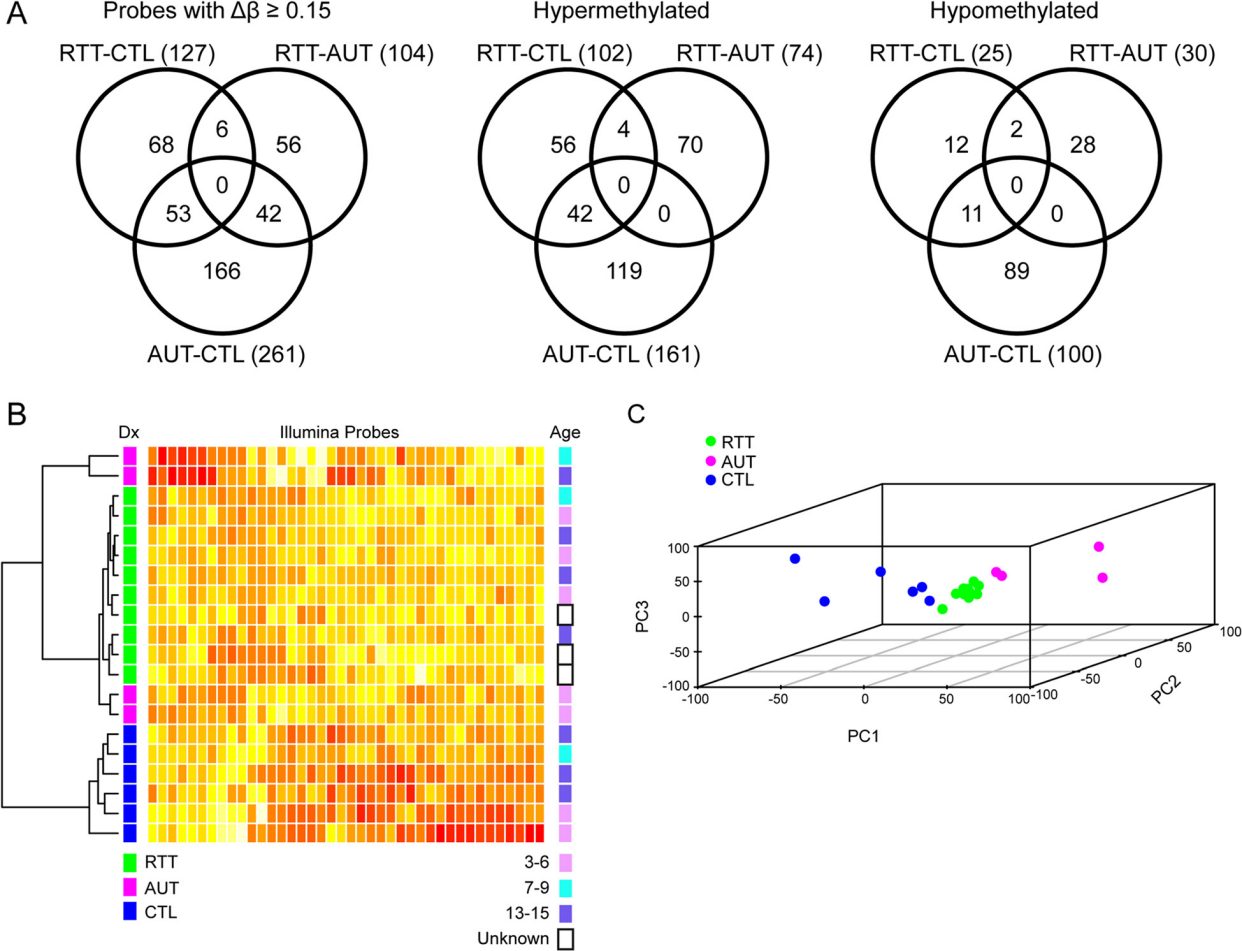
**Probes differentially methylated (Δβ ≥0.15) in neurodevelopmental disorders.** (**A**) Venn diagrams showing 391 probes identified as differentially methylated among Rett syndrome (RTT), autism (AUT), and control (CTL) individuals in the discovery cohort. Probes hypermethylated in RTT and AUT or hypomethylated in RTT and AUT are also shown. (**B**), (**C**) The selected 40 probes comparing lymphoblastoid cell line samples from females diagnosed with neurodevelopmental disorders (RTT, AUT) with CTL females were used for hierarchical clustering and principal component analysis (PCA). (**B**) Hierarchical clustering of the 20 females in the discovery cohort. Each row represents an individual and each column represents one probe. A heat map showing relative methylation differences (yellow = more methylated; red = less methylated) is presented in the clustering dendrogram. The major dendrogram branches defined by the methylation data correspond to diagnosis (Dx), rather than to age. (**C**) PCA of the 40 probe methylation profile for the 20 discovery females. Three principle components (PC1 to PC3), representing 40.7%, 22.1%, and 7.1% of the variance, respectively, are shown.

To identify robust DNA methylation differences (Δβ), we further analyzed all probes with pair-wise mean |Δβ| ≥0.15 among clinical groups within the discovery cohort. This threshold generated a normally distributed dataset containing 391 probes (Additional file 1: Table S2). Among these probes, 127 were differentially methylated between RTT and CTL, 104 between RTT and AUT, and 261 between AUT and CTL (Figure 1A). No probes were differentially methylated among all three pair-wise comparisons, but 53 probes were differentially methylated in either RTT or AUT compared with CTL. Interestingly, 42 probes that were differentially methylated between RTT and AUT and between AUT and CTL were opposite in magnitude. Twenty-six probes hypermethylated in RTT compared with AUT were hypomethylated in AUT compared with CTL, and 16 probes hypomethylated in RTT versus AUT were hypermethylated in AUT versus CTL. The distribution of hypermethylation and hypomethylation loci among comparison pairs was highly significant (χ^2^(2-df) = 14.17, *P* = 0.0008). RTT was hypermethylated compared with CTL and AUT at 102 (80%) and 74 (72%) loci, respectively. AUT was also hypermethylated compared with CTL (62%). The distribution of nonoverlapping loci hypermethylated (72%) or hypomethylated (54%) in AUT compared with CTL was significantly different than loci hypermethylated (82%) or hypomethylated (18%) between RTT and CTL (χ^2^(1-df) = 13.96, *P* = 0.0002).

We performed ANOVA for 391 differentially methylated probes identified in the discovery cohort (Additional file 1: Table S2). Hierarchical clustering using the top 40 significant probes (*P* <0.01, FDR <0.10) suggested discovery samples could be classified based on their DNA methylation profiles (Figure 1B). Principal component analysis further suggested three distinct clusters using the same 40 probes (Figure 1C). DNA methylation among the 40 probes suggested more homogeneity in RTT (mean Pearson’s *r* = 0.93) than among the samples in comparison groups (mean Pearson’s *r* = 0.74 and 0.83 for AUT and CTL groups, respectively).

### Genome-wide CpG DNA methylation: replication cohort

Because seven of the 10 RTT females in this study were reported to have abnormal brain activity (seizures (*n* = 5) or subclinical abnormal EEG activity (*n* = 2)], a common feature of RTT [25], we included an additional subject cohort in the replication experiment. We reasoned that this would address the possibility that AEDs, which can alter DNA methylation [26,27], might underlie some of the differential DNA methylation observed in the discovery cohort. The replication cohort contained LCL samples from females with RTT (*n* = 8), AUT (*n* = 13), SEZ (*n* = 6), or CTL (*n* = 13) (Additional file 1: Table S1). As measured in the discovery cohort, most probes had low DNA methylation levels in these samples (median = 0.06 to 0.12; median = 0.21 to 0.26; range = 0.01 to 0.99). Correlations among samples were high (Pearson’s *r* >0.90), but less similar than samples within the discovery cohort. To ensure technical fidelity across assay runs, replicates from the discovery cohort were included in the DNA methylation analysis of the replication cohort, with nearly perfect correlation between replicate pairs (Pearson’s *r* >0.99) (Additional file 1: Figure S2). The 40 probes that distinguished clinical groups in the discovery cohort analysis similarly distinguished technical replicates, with high correlation between replicate pairs (Pearson’s *r* >0.98) (Additional file 1: Figure S3). Duplicate samples were excluded from the replication cohort analysis.

In the replication cohort, hierarchical clustering using the 40 probes identified in the discovery cohort did not produce clustering of DNA methylation patterns by clinical group (Additional file 1: Figure S4A). Principal component analysis also failed to reveal distinct clinical clusters among the replication samples (Additional file 1: Figure S4B), suggesting greater molecular heterogeneity among the clinical samples included in the replication cohort.

### Locus-specific DNA methylation analysis

To select particular loci with differential DNA methylation among clinical groups, we performed ANOVA for the 40 probes from the discovery cohort analysis using DNA methylation values from the replication cohort samples and in a pooled analysis (Table 1). We then selected probes below the conservative threshold of *P* <0.001 and FDR <0.05 from each analysis. In the discovery cohort, five loci showed significant differential DNA methylation (*HOXA11*, *TCN1*, *PTCD2*, *TAC1*, *ETNK2*). In the replication cohort, no locus was significant. In the pooled analysis, four loci were highly significant (*PTCD2*, *TAC1*, *FLJ44881*, *SPAG7*; *P* <0.0005, FDR <0.005). Overall, no locus emerged across these three analyses. However, *TAC1* differential methylation approached significance in the replication analysis (*P* = 0.003, FDR = 0.13), and was highly significant in both discovery and pooled analyses (*P* <0.0005, FDR <0.05). *TAC1* was previously implicated in RTT pathophysiology through convergent findings in RTT patients and mouse models [28-32]. We thus selected *TAC1* to target for validation.

**Table 1:** Top 40 differentially methylated CpG sites identified in the discovery cohort

		**RTT**		**AUT**		**CTL**		**Discovery (*n*= 20)**		**RTT**		**AUT**		**SEZ**		**CTL**		**Replication (*n*= 40)**		**Pooled (*n*= 60)**	
**ILMN probe**	**Gene**	**Mean β**	**SD**	**Mean β**	**SD**	**Mean β**	**SD**	***P*value**	**FDR**	**Mean β**	**SD**	**Mean β**	**SD**	**Mean β**	**SD**	**Mean β**	**SD**	***P*value**	**FDR**	***P*value**	**FDR**
cg17950095	*HOXA11*	0.25	0.06	0.20	0.02	0.37	0.07	**0.0002**	**0.03**	0.23	0.12	0.27	0.11	0.27	0.09	0.25	0.12	0.86	0.92	0.42	0.46
cg00187686	*TCN1*	0.38	0.07	0.41	0.13	0.18	0.07	**0.0002**	**0.03**	0.48	0.14	0.40	0.09	0.43	0.05	0.37	0.06	0.05	0.32	0.003	0.02
cg04527989	*PTCD2*	0.83	0.04	0.64	0.11	0.83	0.06	**0.0003**	**0.03**	0.73	0.09	0.70	0.07	0.64	0.16	0.77	0.05	0.03	0.32	**0.0001**	**0.001**
cg14221171	*TAC1*	0.17	0.09	0.35	0.08	0.10	0.06	**0.0004**	**0.03**	0.23	0.11	0.22	0.08	0.35	0.11	0.16	0.08	0.003	0.13	**0.0001**	**0.001**
cg03718539	*ETNK2*	0.07	0.03	0.24	0.05	0.14	0.10	**0.0004**	**0.03**	0.11	0.03	0.12	0.06	0.16	0.06	0.09	0.04	0.07	0.32	0.02	0.05
cg09242541	*APITD1*	0.53	0.14	0.45	0.11	0.24	0.07	0.001	0.04	0.41	0.22	0.43	0.16	0.32	0.08	0.54	0.13	0.04	0.32	0.28	0.34
cg18432105	*MYH2*	0.74	0.06	0.62	0.03	0.57	0.11	0.001	0.06	0.66	0.09	0.70	0.07	0.71	0.03	0.67	0.13	0.62	0.81	0.17	0.24
cg14986136	*WBP5*	0.13	0.08	0.13	0.07	0.32	0.10	0.001	0.06	0.21	0.14	0.17	0.08	0.20	0.05	0.19	0.11	0.77	0.90	0.17	0.24
cg18838701	*TNNI3*	0.14	0.07	0.34	0.20	0.08	0.05	0.002	0.06	0.26	0.14	0.24	0.14	0.23	0.10	0.21	0.12	0.87	0.92	0.15	0.22
cg06537230	*DLX5*	0.29	0.09	0.35	0.05	0.16	0.06	0.002	0.06	0.27	0.09	0.30	0.08	0.37	0.09	0.29	0.09	0.20	0.65	0.02	0.05
cg19002579	*SMPX*	0.64	0.09	0.69	0.04	0.42	0.17	0.002	0.06	0.64	0.12	0.61	0.08	0.66	0.08	0.60	0.09	0.54	0.78	0.02	0.05
cg06618866	*TLR2*	0.16	0.05	0.26	0.09	0.10	0.05	0.002	0.06	0.16	0.08	0.18	0.09	0.16	0.05	0.14	0.05	0.53	0.78	0.02	0.05
cg23196831	*COL14A1*	0.22	0.05	0.25	0.12	0.09	0.06	0.002	0.06	0.18	0.11	0.25	0.10	0.23	0.11	0.18	0.09	0.26	0.67	0.02	0.05
cg01541443	*C7orf41*	0.78	0.07	0.61	0.10	0.79	0.05	0.002	0.06	0.67	0.10	0.67	0.08	0.68	0.03	0.66	0.17	0.99	0.99	0.33	0.38
cg19642007	*TNNT3*	0.62	0.06	0.45	0.11	0.47	0.09	0.003	0.06	0.49	0.12	0.49	0.07	0.53	0.09	0.47	0.07	0.57	0.79	0.02	0.05
cg00176210	*ANK1*	0.37	0.09	0.42	0.05	0.22	0.10	0.003	0.06	0.40	0.11	0.34	0.12	0.41	0.06	0.34	0.10	0.39	0.68	0.05	0.09
cg02049180	*INSRR*	0.65	0.07	0.43	0.19	0.64	0.03	0.003	0.06	0.62	0.11	0.62	0.07	0.64	0.04	0.61	0.09	0.92	0.95	0.23	0.31
cg09868035	*C20orf135*	0.45	0.07	0.38	0.11	0.29	0.07	0.003	0.07	0.45	0.10	0.44	0.13	0.41	0.07	0.38	0.12	0.49	0.78	0.04	0.09
cg26227465	*IFNG*	0.69	0.07	0.74	0.09	0.49	0.17	0.003	0.07	0.58	0.15	0.60	0.13	0.60	0.14	0.55	0.19	0.79	0.90	0.08	0.13
cg20322862	*TGIF1*	0.51	0.07	0.37	0.07	0.56	0.08	0.003	0.07	0.46	0.11	0.51	0.08	0.53	0.05	0.45	0.13	0.30	0.68	0.79	0.81
cg09404633	*LMOD1*	0.35	0.08	0.39	0.11	0.16	0.14	0.004	0.07	0.30	0.12	0.28	0.11	0.37	0.08	0.25	0.10	0.13	0.48	0.01	0.03
cg04555771	*CACNA2D2*	0.33	0.10	0.44	0.10	0.16	0.14	0.004	0.07	0.28	0.08	0.32	0.11	0.37	0.06	0.36	0.15	0.39	0.68	0.40	0.45
cg10467098	*C11orf68*	0.47	0.08	0.33	0.09	0.53	0.08	0.005	0.08	0.40	0.11	0.48	0.09	0.44	0.09	0.49	0.14	0.22	0.67	0.21	0.29
cg04091078	*SLCO1C1*	0.73	0.04	0.58	0.14	0.71	0.04	0.005	0.09	0.68	0.07	0.67	0.08	0.71	0.04	0.67	0.05	0.50	0.78	0.05	0.09
cg05570980	*C3orf52*	0.19	0.05	0.31	0.13	0.14	0.05	0.006	0.09	0.16	0.03	0.17	0.06	0.18	0.06	0.22	0.10	0.24	0.67	0.71	0.75
cg02441647	*COL8A1*	0.08	0.03	0.13	0.04	0.25	0.15	0.006	0.09	0.17	0.09	0.14	0.06	0.17	0.07	0.15	0.12	0.86	0.92	0.25	0.32
cg18801691	*DCC*	0.12	0.05	0.07	0.03	0.24	0.13	0.007	0.09	0.26	0.11	0.18	0.05	0.23	0.05	0.17	0.06	0.03	0.32	0.28	0.34
cg21306775	*FLJ44881*	0.71	0.05	0.57	0.17	0.50	0.16	0.007	0.09	0.65	0.12	0.60	0.10	0.54	0.05	0.53	0.10	0.07	0.32	**0.0002**	**0.002**
cg14141399	*HAS1*	0.89	0.03	0.79	0.16	0.69	0.16	0.007	0.09	0.76	0.15	0.81	0.05	0.76	0.09	0.75	0.12	0.52	0.78	0.05	0.09
cg12815142	*SPAG7*	0.69	0.05	0.56	0.06	0.53	0.15	0.007	0.09	0.58	0.09	0.53	0.08	0.60	0.04	0.51	0.10	0.07	0.32	**0.0003**	**0.003**
cg14062083	*KRTAP13-4*	0.56	0.08	0.42	0.13	0.39	0.10	0.007	0.09	0.46	0.10	0.45	0.09	0.57	0.04	0.44	0.12	0.05	0.32	*0.003*	*0.02*
cg20311730	*NLRP10*	0.64	0.04	0.60	0.11	0.47	0.14	0.007	0.09	0.59	0.10	0.58	0.05	0.60	0.05	0.54	0.08	0.34	0.68	*0.004*	*0.02*
cg00415993	*F2RL2*	0.67	0.10	0.65	0.11	0.44	0.18	0.008	0.09	0.58	0.08	0.51	0.13	0.59	0.07	0.44	0.19	0.10	0.42	*0.002*	*0.01*
cg00318573	*CHRNA4*	0.32	0.12	0.18	0.09	0.14	0.07	0.008	0.09	0.20	0.08	0.19	0.11	0.25	0.12	0.16	0.09	0.32	0.68	0.01	0.03
cg15350036	*CROT*	0.82	0.04	0.75	0.07	0.66	0.13	0.008	0.09	0.78	0.04	0.72	0.09	0.73	0.08	0.67	0.20	0.35	0.68	0.01	0.04
cg04993257	*PLAC2*	0.44	0.07	0.35	0.08	0.26	0.15	0.009	0.09	0.39	0.07	0.41	0.10	0.44	0.07	0.47	0.10	0.27	0.67	0.82	0.82
cg03273615	*RBM41*	0.40	0.08	0.33	0.12	0.22	0.11	0.009	0.09	0.35	0.12	0.35	0.10	0.38	0.04	0.32	0.13	0.73	0.88	0.08	0.13
cg11505048	*APOBEC4*	0.83	0.06	0.72	0.14	0.88	0.03	0.009	0.09	0.71	0.09	0.69	0.07	0.69	0.07	0.73	0.08	0.60	0.80	0.01	0.04
cg01145396	*CHRNG*	0.58	0.07	0.57	0.08	0.43	0.12	0.009	0.09	0.49	0.07	0.49	0.08	0.57	0.06	0.52	0.14	0.36	0.68	0.32	0.37
cg13370916	*STARD8*	0.58	0.06	0.51	0.02	0.40	0.16	0.009	0.09	0.48	0.10	0.50	0.09	0.51	0.05	0.47	0.10	0.70	0.87	0.04	0.09

Probes with *P* <0.001 and FDR <0.05 are in bold. Probes with P <0.01 and FDR <0.05 are in italic. *AUT* autism, *CTL* control, *FDR* false discovery rate, *ILMN Probe* Illumina HumanMethyl27 probe name; *P value* analysis of variance *P* value, *RTT* Rett syndrome, *SD* standard deviation.

**Figure 2:**
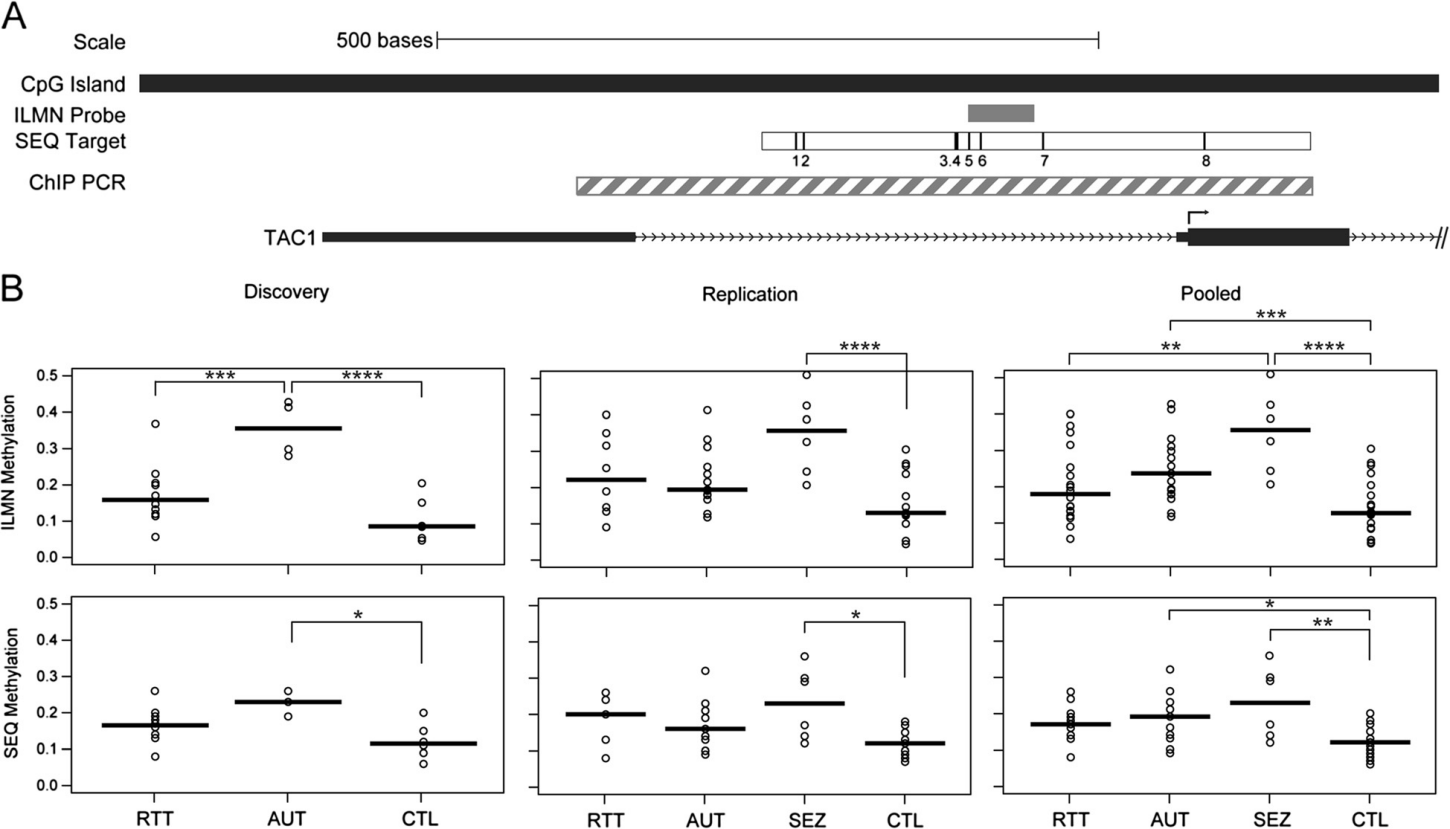
**Physical map of the *TAC1 *promoter region and differential DNA methylation in lymphoblastoid cell lines.** (**A**) *TAC1* is shown 5′ → 3′ on the positive strand (chr7:97,199,050 to 97,200,050). Direction of gene transcription (arrowheads) and transcription start site (TSS; arrow) are indicated. The differentially methylated region overlaps with a large CpG island that includes the TSS. CpG Island Track from UCSC Human Genome Browser hg18 (black bar); Illumina (ILMN) probe (grey bar); Sequenom (SEQ) target region (white bar) with CpGs (horizontal lines); chromatin immunoprecipitation (ChIP) PCR amplicon (striped bar). Locus diagram is drawn to scale. (**B**) Tukey *post-hoc* pair-wise comparisons of *TAC1* DNA methylation (%) assayed using ILMN (cg14221171) and validated using SEQ (CpG site 5). DNA methylation for each sample (circles) and group mean (horizontal bar) are shown for each clinical group. *P <0.05, **P <0.01, ***P <0.005. AUT, autism; CTL, control; RTT, Rett syndrome; SEZ, seizure disorder.

A Sequenom EpiTYPER assay was designed to quantify DNA methylation within the *TAC1* promoter CpG island (Figure 2A). First, we determined whether DNA methylation levels detected at specific CpG sites within the Sequenom target region validated the Illumina assay results. Sequenom DNA methylation levels at two CpG sites located closest to the Illumina probe were highly correlated within samples (Additional file 1: Figure S5). Next, we evaluated whether significant differential methylation occurred among clinical groups using the Sequenom CpG methylation values. The clinical group had a significant main effect on DNA methylation at *TAC1* CpG site 5 (ANOVA *F*_(3,52)_ = 5.70, *P* = 0.002). DNA methylation levels at this site had the strongest correlation with Illumina probe methylation values and validated the *TAC1* results obtained from the genome-wide analysis.

To determine which clinical groups exhibited *TAC1* differential methylation, we performed Tukey *post-hoc* pair-wise comparisons for discovery, replication, and pooled cohorts using Illumina and Sequenom data (Figure 2B). In the Illumina dataset, *TAC1* was hypomethylated in RTT compared with AUT (*P* = 0.003) in the discovery cohort, and compared with SEZ (*P* = 0.008) in the pooled analysis. AUT was significantly hypermethylated relative to CTL in discovery (*P* = 0.0003) and pooled analyses (*P* = 0.004). SEZ was significantly hypermethylated versus CTL in replication (*P* = 0.001) and pooled analyses (*P* = 0.0001). Sequenom data confirmed significant AUT hypermethylation versus CTL in discovery (*P* = 0.02) and pooled analyses (*P* = 0.04), and SEZ hypermethylation versus CTL in replication (*P* = 0.01) and pooled analyses (*P* = 0.002). Overall, SEZ hypermethylation relative to CTL emerged with the most significant differential DNA methylation in both Illumina and Sequenom datasets.

### TAC1 expression in brain

**Figure 3:**
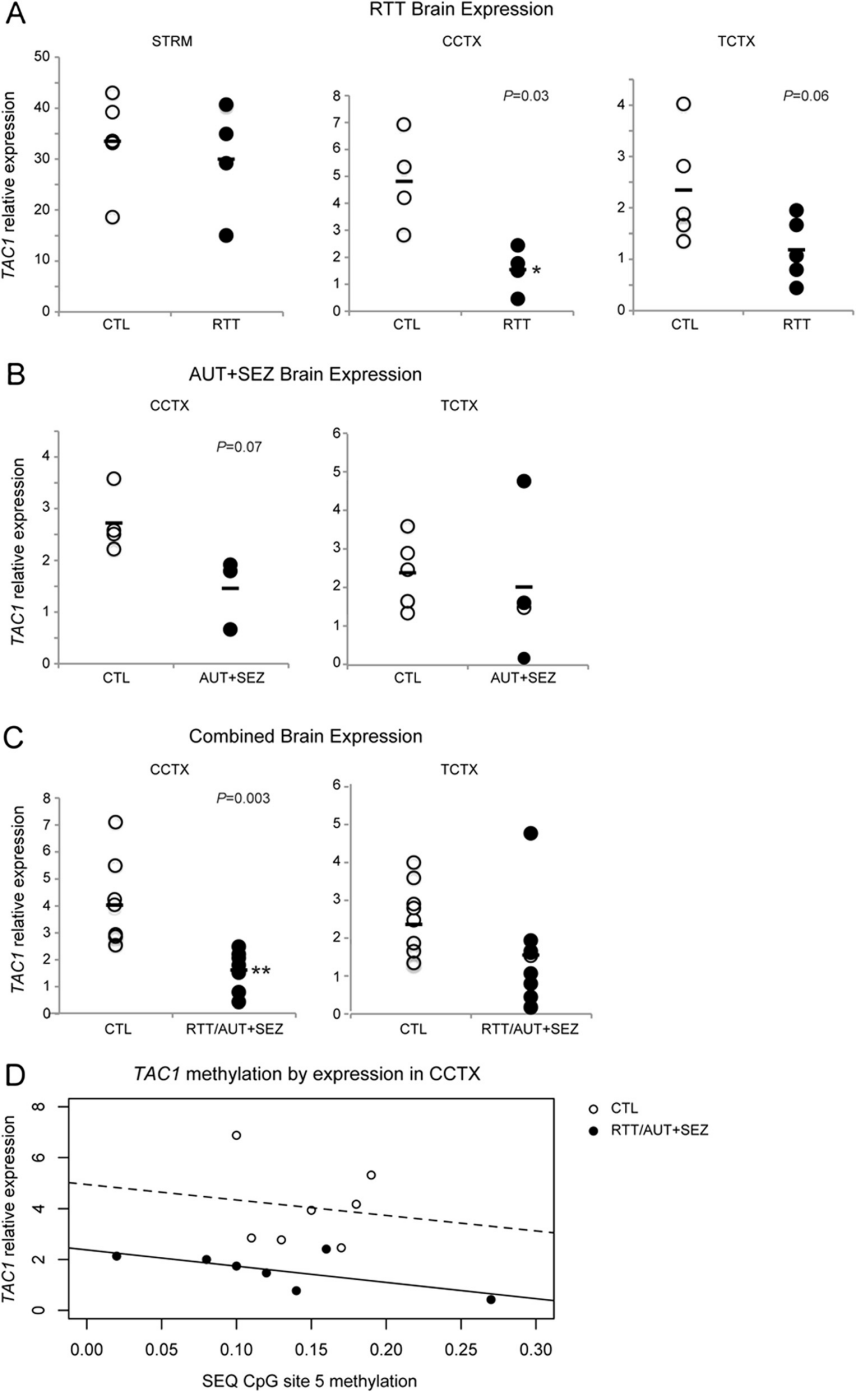
***TAC1 *expression and DNA methylation in the brain.** (**A**), (**B**), (**C**) *TAC1* mRNA assayed by quantitative RT-PCR in the striatum (STR), cingulate (CCTX) and temporal cortex (TCTX) of female brains. Data are presented as relative expression of *TAC1* compared with *ACTB* for each sample (circles) and group mean (horizontal bar). *P <0.05; **P <0.01. (**D**) Scatter plot of DNA methylation at Sequenom (SEQ) CpG site 5 and *TAC1* expression in the CCTX stratified by seizure phenotype (RTT/AUT+SEZ) or control (CTL). Regression lines for seizure phenotype (black) and CTL (dashed) indicate an inverse relationship between DNA methylation and expression in CCTX. AUT, autism; RTT, Rett syndrome; SEZ, seizure disorder.

Since DNA methylation at CpG islands proximal to gene promoter regions is an important mediator of gene transcription [33], we evaluated whether *TAC1* promoter DNA methylation differences were associated with differential expression. Because *TAC1* is only weakly expressed in lymphocytes [34,35], evaluating expression differences in LCLs among clinical groups was uninformative (Additional file 1: Figure S6). However, if altered DNA methylation observed in LCLs occurred early in development, then differential DNA methylation detected in LCLs might exist in additional tissues. *TAC1* is expressed in brain throughout development [36,37], and the brain is the primary tissue affected in neurodevelopmental disorders. We thus examined whether *TAC1* DNA methylation status from a peripherally derived tissue correlates with central gene expression. DNA methylation analysis revealed significant *TAC1* hypermethylation in SEZ versus CTL. Given that seizures commonly occur in RTT [25], we obtained postmortem brain samples from RTT females. *TAC1* expression was assayed in three brain regions from age-matched RTT and CTL females (Additional file 1: Table S3) using quantitative RT-PCR (Figure 3A). *TAC1* expression was significantly reduced in RTT cingulate cortex (*t*(6) = 2.98, *P* = 0.03), with a trend for reduced expression in temporal cortex (*t*(8) = 2.22, *P* = 0.06). No differences in *TAC1* expression were detected in striatum (*t*(7) = 0.54, *P* = 0.61). Given these results, we examined *TAC1* expression in additional postmortem brain samples obtained from female subjects diagnosed with AUT and for which the patient’s cause of death was ascribed to SEZ [38] (Figure 3B). Indeed, *TAC1* expression was also reduced in AUT+SEZ cingulate cortex, approaching significance (*t*(5) = 2.29, *P* = 0.07). No significant difference in *TAC1* expression was detected in AUT temporal cortex (*t*(7) = 0.98, *P* = 0.36). A combined analysis using all brain samples and comparing *TAC1* expression in the cingulate cortex suggested a significant *TAC1* reduction in affected brains (*t*(12) = 3.67, *P* = 0.003) (Figure 3C).

### TAC1 DNA methylation in brain

Several studies have reported differential DNA methylation among brain regions in postmortem tissue [39,40]. To determine whether DNA methylation at the *TAC1* promoter varied among brain regions, we selected Illumina DNA methylation data (cg14224417) assayed in four brain regions from female samples in a previous study [39]. The *TAC1* promoter displayed significant differential DNA methylation among certain brain regions in females (Additional file 1: Figure S7).

To determine whether the reduced *TAC1* expression observed in cingulate cortex of seizure phenotype (RTT and AUT+SEZ) brains may be associated with differential DNA methylation, we extracted DNA from the same brain samples used for the expression studies and evaluated *TAC1* DNA methylation using the Sequenom EpiTYPER assay. As expected, an inverse correlation between *TAC1* promoter DNA methylation and expression was observed for both seizure phenotype and CTL cingulate cortex samples (Figure 3D). However, no significant differential DNA methylation was detected at *TAC1* CpG site 5 between seizure phenotype and CTL cingulate cortex samples (*t*(16) = −0.71, *P* = 0.49), nor for any of the other CpG sites in the *TAC1* promoter locus (data not shown). DNA methylation at *TAC1* CpG site 5 was also not a significant predictor of *TAC1* expression in the cingulate cortex of CTL brains (β = −6.09, *P* = 0.78). However, in this small sample, there was a trend suggesting DNA methylation at *TAC1* CpG site 5 may predict *TAC1* expression in the cingulate cortex of seizure phenotype brains (β = −6.40, *P* = 0.09).

### MeCP2 binds to the TAC1 promoter

**Figure 4:**
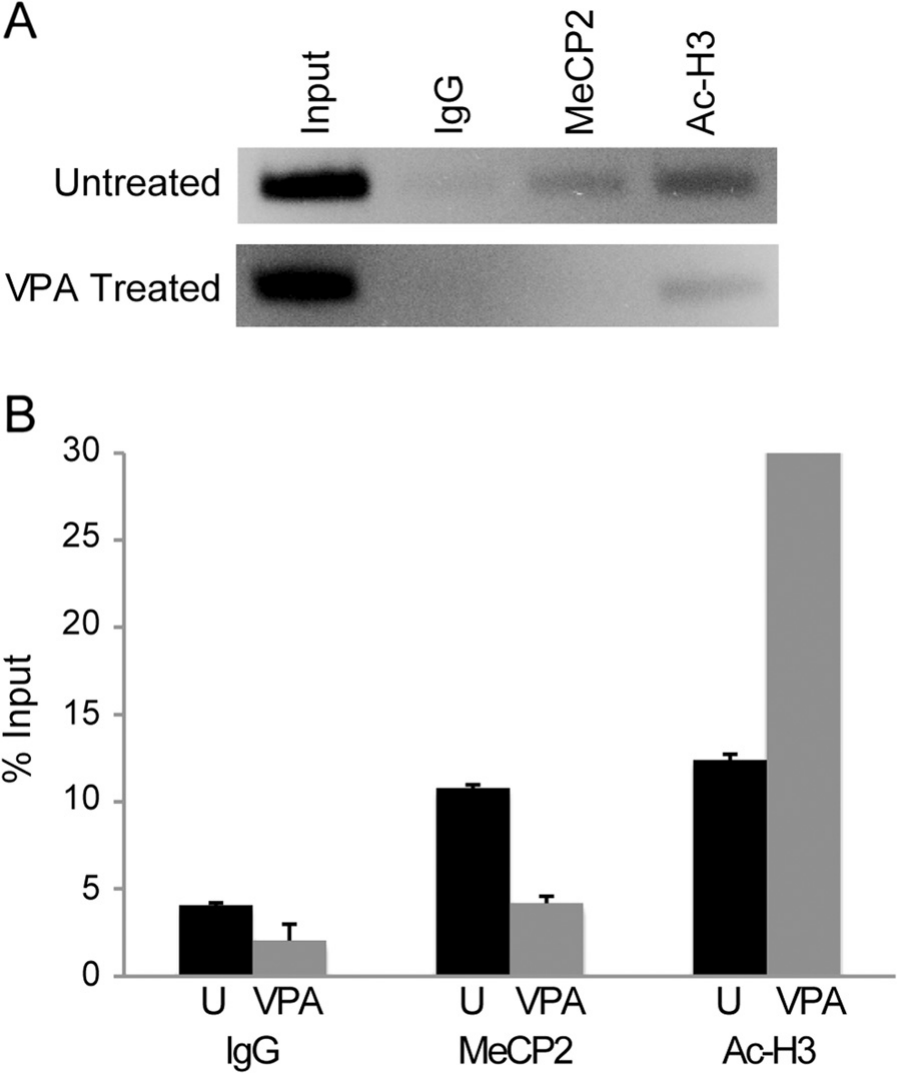
**MeCP2 binding to the *TAC1 *promoter *in vivo*.** (**A**) Chromatin immunoprecipitation(ChIP) assay in untreated and 3 mM valproic acid (VPA)-treated HEK cells. DNA fragments immunoprecipitated with IgG, anti-MeCP2 or anti-acetyl histone H3 (Ac-H3) were analyzed by PCR with primers specific for the *TAC1* promoter locus (Figure 2A). (**B**) Reduced MeCP2 and increased Ac-H3 occupancy at the *TAC1* promoter in DNA extracted from untreated (black) or VPA-treated (grey) HEK cells, assayed in triplicate by quantitative PCR analysis of ChIP samples. Ac-H3 quantification extends beyond the graph to 105% input.

RTT is mostly caused by *MECP2* coding mutations [6]. More recently, increased *MECP2* expression was detected in temporal cortex of patients with intractable temporal lobe epilepsy [41]. To determine whether *TAC1* is a MeCP2 target *in vivo*, we performed chromatin immunoprecipitation assays in HEK cells, which express high levels of *TAC1*[34,35]. Anti-MeCP2 specifically precipitates *TAC1* DNA, while control IgG yields no enrichment of binding to the *TAC1* sequence (Figure 4A), supporting direct binding of MeCP2 to the *TAC1* promoter region (Figure 2A). We further examined the possibility that DNA hypermethylation detected at the *TAC1* promoter in seizure-associated phenotypes was due to AED treatment. VPA is an anticonvulsant, commonly administered for seizure control in RTT, and a potent histone deacetylase inhibitor that can directly alter DNA methylation *in vitro*[26]. Following VPA treatment of HEK cells, anti-MeCP2 failed to precipitate *TAC1* DNA, whereas anti-acetylated histone 3 precipitates more DNA, as expected (Figure 4A, B).

## Discussion

The present study demonstrates both the challenges and potential promise of utilizing genome-wide DNA methylation patterns in LCLs derived from peripheral cells to distinguish individuals with different neurodevelopmental disorders. An initial analysis of LCLs from CTLs and the neurodevelopmental disorders RTT and AUT was sufficiently specific to cluster each cohort. However, a larger replication sample did not repeat this finding. Importantly, the additional subject analyses revealed that secondary clinical complications that can accompany many neurodevelopmental disorders, such as SEZ, may have significant effects on DNA methylation status. In contrast to diseases like cancer [42], alterations in genome-wide DNA methylation patterns in neurodevelopmental disorders are likely to be subtle. We therefore suggest that utilizing samples from patients with detailed clinical and molecular information on each subject included in such studies will be required to discover unique disorder-specific DNA methylation patterns that will be useful for sorting clinical heterogeneity within and across disorders.

Analysis across all clinical groups suggested differential methylation of the *TAC1* gene in LCLs derived from peripheral cells. This turned out to be highly relevant with regard to RTT, given that it is one of the few genes whose expression is consistently reduced in the brains of *Mecp2* mutant mice [31,32]. Our study is the first to examine *TAC1* expression in postmortem brain samples from RTT subjects. We demonstrate reduced *TAC1* expression in brain samples from subjects defined by clinical diagnosis and presence of seizures. The principle protein responsible for DNA methylation-dependent transcriptional regulation is MeCP2, encoded by a gene mutated in RTT. Further analysis showed direct MeCP2–*TAC1* promoter binding, but also suggested that the interaction is highly sensitive to the AED VPA *in vitro*. Collectively, these findings reflect the complexity of utilizing epigenetic differences in categorical analyses based on a clinical diagnosis. The challenges and utility of the approaches used here are discussed below.

### Challenges of clinical stratification using differential DNA methylation patterns

DNA methylation is one of several key mechanisms through which gene expression is controlled. Analysis of DNA methylation patterns has been the focus of comparative studies in normal and malignant tissues [43], biopsied samples from peripheral organs [44-46], and in white blood cells from control and disorder-diagnosed populations [21,47,48]. This strategy is a challenge particularly with neurological disorders, because molecular assays of brain are often not possible in patients. Several studies have examined DNA methylation patterns in postmortem brain tissue from control subjects and those with a psychiatric disorder diagnosis [49,50], but the relationship between peripheral and central measures of DNA methylation are only beginning to emerge [45]. Moreover, there is variability in the fidelity of different assays used in studies, and few studies use secondary methods to validate alternations in specific DNA methylation sites. The 27K chip has been shown to be highly reliable and sensitive across assay runs [19,23], and we showed in the present study that replicate samples run at different times, independent of diagnostic group, are >99% identical in terms of global DNA methylation patterns. One caveat of our study was the use of the 27K chip, the only validated chip at the time we initiated the studies. This chip has high fidelity and samples CpG sites across the genome, although it only probes approximately one-half of the genes in the genome. Future studies using assays with greater coverage will therefore possibly reveal new patterns of DNA methylation not recognized here. An additional technical caveat is the use of patient LCLs. These cells provide an opportunity to assess patient-derived samples that are commonly accumulated in repositories in sufficient numbers for the study of rare neurological disorders. However, the transformation and growth processes of these cell lines may lead to epigenetic changes not present in the primary patient cells [51]. These challenges notwithstanding, the present study emphasizes the importance of controlling for complex clinical phenotypes, such as seizures. The data presented here suggest that the state-dependent status of DNA methylation patterns, as well as central and peripheral differences, may make it challenging to detect disorder-specific patterns that consistently reflect categorical differences across samples.

### DNA methylation in Rett syndrome

We initially focused on RTT due to the importance of MeCP2 in regulating transcription through binding to methylated DNA. The possibility of altered DNA methylation in RTT had not previously been investigated in clinical samples. We hypothesized that the altered MeCP2 function in RTT might lead to compensatory changes in the DNA methylation status of certain genes. In fact, the initial sample illustrated that using LCLs from patients, analyzed using cluster and principle component methods of the top differentially methylated genes, distinguished the clinical groups from CTL. More than double the number of CpG sites showed differential DNA methylation between AUT and CTL (*n* = 261) than between RTT and CTL (*n* = 127). At first glance, these results were surprising given our hypothesis that RTT would display the greatest differential DNA methylation from CTL because of the involvement of MeCP2 in this disorder. However, the more limited differences in the first RTT cohort are not unprecedented. For example, in another neurodevelopmental disorder, Cornelia de Lange syndrome, fewer CpG sites demonstrated differential methylation than the number of genes with expression differences [52,53]. Expression profiling in AUT has been more compelling than in RTT. Hundreds of differentially expressed genes in LCLs and brain from AUT subjects have been reported [54,55], while global gene expression analyses have failed to reveal any dramatic differences in RTT compared with control subjects using a variety of tissues [56-61]. The present data showing an inverse correlation between DNA methylation and *TAC1* expression, despite the reduced *TAC1* expression in cortex, may be a reflection of the small sample size and/or the complex relationship between transcriptional regulation and gene expression.

The initial promise of categorizing clinical groups by DNA methylation pattern from the first cohort did not replicate in a second, larger sample that included LCLs from individuals with SEZ. DNA methylation status is highly influenced by nonheritable factors, including physiological state [62], medications [26] and even diet [63]. We reasoned that since the presence of seizures in neurodevelopmental disorders is common and a hallmark of RTT, it could serve as a unifying feature in the clinically diagnosed populations. In fact, using LCLs from subjects with SEZ and postmortem brain tissue from AUT cases in which seizures were noted in the medical history, or was the cause of death, we show that DNA methylation patterns of specific genes may be more related to seizure status, a finding that has not been previously reported. The repositories that provided the LCLs and postmortem brain samples do not collect sufficient information regarding medication status. We can therefore only suggest that contributions to DNA methylation status at certain sites may be due in part to drug treatment effects. To investigate this possibility, VPA-treated HEK cells showed dramatic changes in DNA methylation of the *TAC1* gene; additional sites were probably also impacted by VPA. Interestingly, VPA treatment altered MeCP2 binding to the *TAC1* promoter, which suggests that this putative regulatory interaction may be responsible in part for the consistent reduction of *TAC1* expression in cortical brain regions that we assayed, although not in striatum.

### *TAC1* as a target gene in Rett syndrome

The *TAC1* DNA methylation and gene expression changes were not expected, although this gene has been a candidate target in RTT due to the finding of reduced expression in *Mecp2* mutant mice [31,32]. The present study is the first to identify molecular changes of *TAC1* in brain samples from RTT patients. *TAC1* gene expression changes were statistically significant in the cingulate cortex, with a trend that almost reached significance in the temporal cortex given the limited sample size; other brain regions should be evaluated in the future. It is noteworthy that *TAC1* expression changes were not observed in the striatum, indicating that seizure pathophysiology and AEDs do not produce global, nonspecific effects throughout the brain. The alterations in the cingulate cortex are of functional interest, due to the role of this cortical region in executive function and complex multisensory processing [64-66], both of which are disrupted in AUT and SEZ [67,68]. The *TAC1* gene encodes neurokinins and substance P. These neuromodulators are expressed in nociceptive primary sensory neurons, and serve as modulators of pain perception and inflammation [69]. In the neocortex, substance P is an excitatory neuromodulator of projection neuron activity [70]. Furthermore, there are several convergent findings related to expression of this peptide and RTT. First, substance P is reduced in the cerebrospinal fluid of RTT patients [28]. Second, substance P immunoreactivity is reduced in brains of RTT patients compared with age-matched controls [30], although not in the bowels (enteric nervous system) [29]. Third, *Tac1* expression is reduced in the hypothalamus of juvenile male *Mecp2* mutant mice [31] and in adult male mice with postnatal *Mecp2* loss [32]. Finally, respiratory abnormalities are a clinical hallmark of RTT [71] and neurons in the rhythm-generating center of the respiratory network are dynamically regulated by substance P [72]. The discovery of *TAC1* deficiency in RTT thus adds to other convergent lines of evidence implicating the neuropeptides in RTT pathophysiology. Further consideration of substance P and other neuropeptides transcribed from the *TAC1* locus as therapeutic targets in RTT and AUT+SEZ is warranted.

## Conclusion

We have examined genome-wide CpG DNA methylation patterns in LCLs from females with three related neurodevelopmental disorders. The initial analysis suggested promise of differential DNA methylation patterns among clinical disorders, but failure to replicate this finding in a second, larger cohort may reflect a more complex relationship between DNA methylation status and neurological disorder. This may be due to the presence of seizures in neurodevelopmental disorders and/or the use of medications that can affect DNA methylation, such as VPA. Currently, there is a limited understanding of the complex relationship between DNA methylation status of peripheral cells and brain tissues. Interestingly, we found that hypermethylation at the *TAC1* promoter in LCLs was correlated with reduced expression in certain brain regions, despite the lack of a change in DNA methylation of the *TAC1* promoter in the brain. Further, we show that MeCP2 directly binds to the *TAC1* promoter *in vivo*, and this binding can be altered dramatically by AED treatment. These findings suggest that future studies with larger sample cohorts should consider shared co-occurring clinical features, such as seizures, as well as specific drug treatments in order to more thoroughly address the role of altered DNA methylation in neurodevelopmental disorders.

## Availability of supporting data

These data have been deposited into the NCBI Gene Expression Omnibus [73] and are accessible through GEO Series accession number GSE345099.

## Abbreviations

AED: Antiepileptic drug; ANOVA: Analysis of variance; AUT: Autism; CTL: Control; FDR: False discovery rate; LCL: Lymphoblastoid cell line; PCR: Polymerase chain reaction; RT: Reverse transcriptase; RTT: Rett syndrome; SEZ: Seizure disorder; SNP: Single nucleotide polymorphism; VPA: Valproic acid.

## Competing interests

The authors declare that they have no competing interests.

## Authors’ contributions

KAA and PL designed the study. KAA performed experiments and analyzed data. JTP performed experiments. KAA and PL drafted the manuscript. All authors read, edited and approved the final manuscript.

## Supplementary Material

Additional file 1 A pdf file containing supplementary methods, Figures S1 to S7 and Tables S1 to S5. Click here for file
