# Supplementary material for: Comparative DNA methylation among females with neurodevelopmental disorders and seizures identifies TAC1 as a MeCP2 target gene
Source: J Neurodev Disord. 2013 Jun 11;5(1):15. doi: 10.1186/1866-1955-5-15 (PMC3700820; doi:10.1186/1866-1955-5-15)
Supplement: Additional file 1 — A pdf file containing supplementary methods, Figures S1 to S7 and Tables S1 to S5. [file 1866-1955-5-15-S1.pdf]

Comparative DNA methylation among females with neurodevelopmental disorders and seizures identifies *TAC1* as a MeCP2 target gene.

Kimberly A. Aldinger[1], Jasmine T. Plummer[1], Pat Levitt[1,2]

[1] Zilkha Neurogenetic Institute, Keck School of Medicine of USC, Los Angeles, CA 90089;
[2] Department of Cell & Neurobiology, Keck School of Medicine of USC, Los Angeles, CA 90089

Supplementary Materials:
Figures S1–7
Tables S1–5

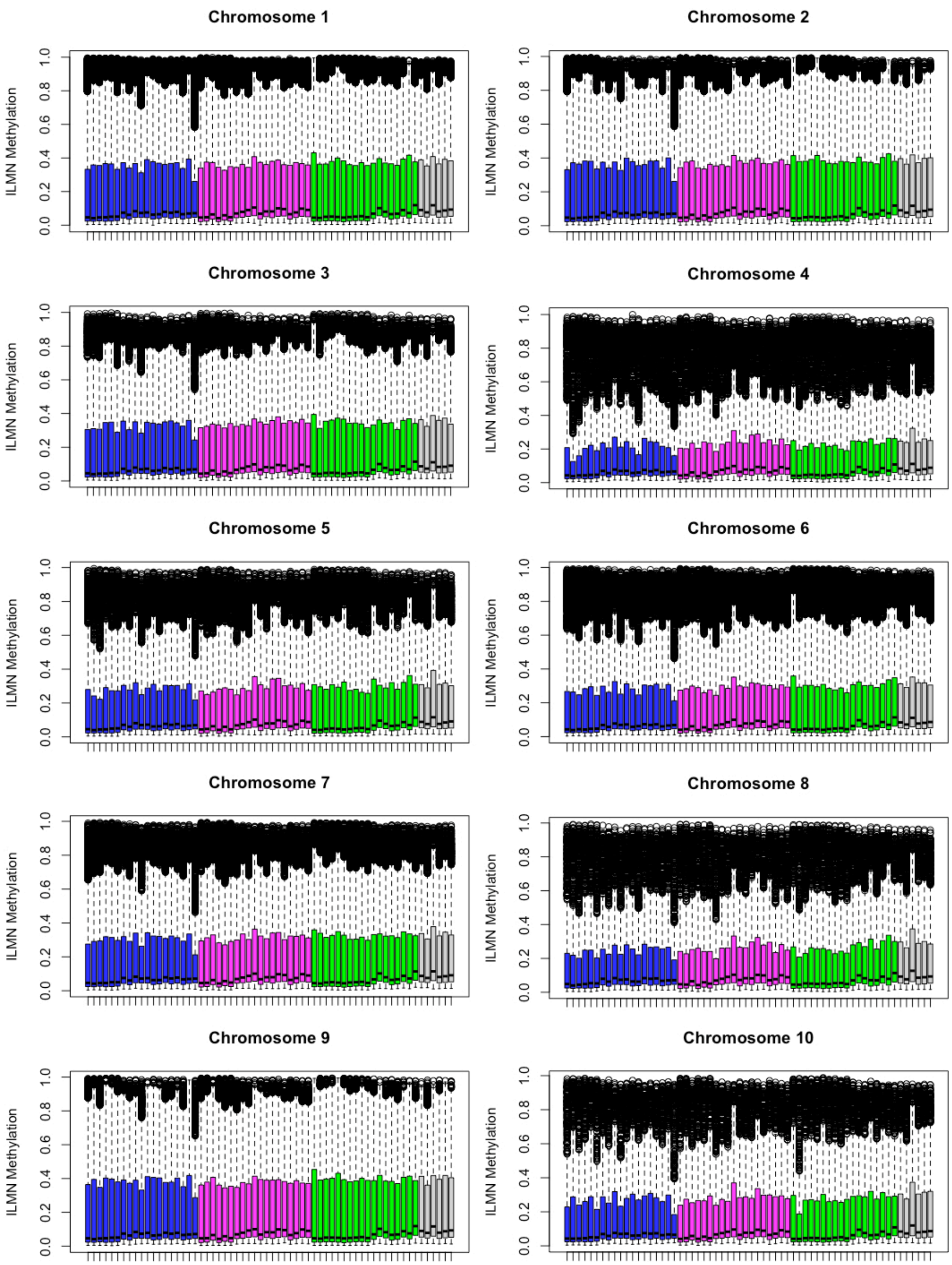
Chromosome 1
Chromosome 2
Chromosome 3
Chromosome 4
Chromosome 5
Chromosome 6
Chromosome 7
Chromosome 8
Chromosome 9
Chromosome 10
ILMN Methylation
0.0
0.2
0.4
0.6
0.8
1.0

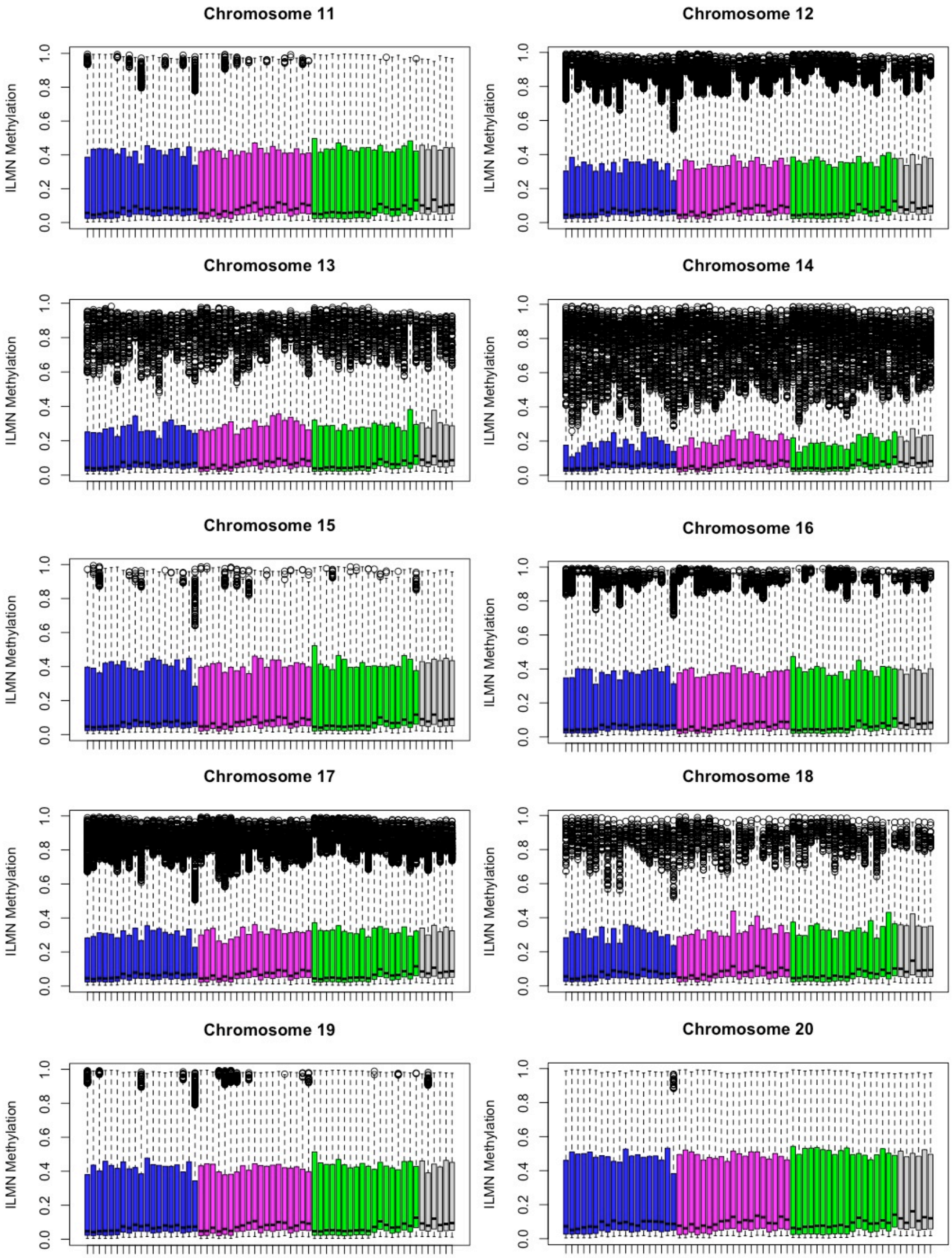
Chromosome 11
Chromosome 12
Chromosome 13
Chromosome 14
Chromosome 15
Chromosome 16
Chromosome 17
Chromosome 18
Chromosome 19
Chromosome 20
ILMN Methylation
0.0
0.2
0.4
0.6
0.8
1.0

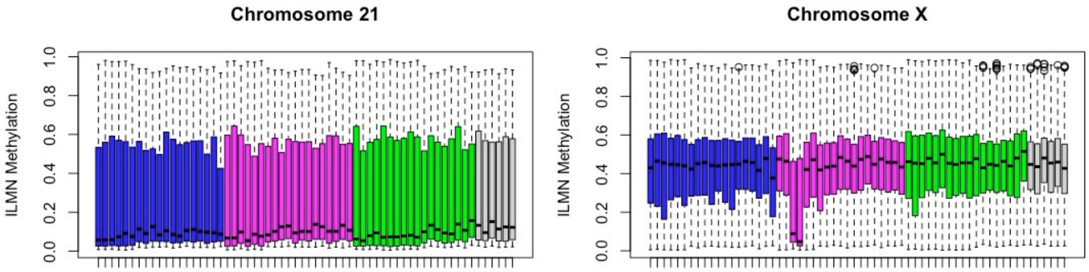

Figure S1. DNA methylation per chromosome. Boxplots of DNA methylation for each individual are presented (blue = CTL; pink = AUT, green = RTT, grey = SEZ). Two AUT samples display skewed DNA methylation for chromosome X. SRY+ genotyping confirmed the sex discrepancy in these samples (data not shown). No other chromosome abnormalities were apparent. Copy number variant (CNV) analysis was previously reported for 11 samples (1, 2). Reported CNVs are listed in **Supplemental Table 4.**

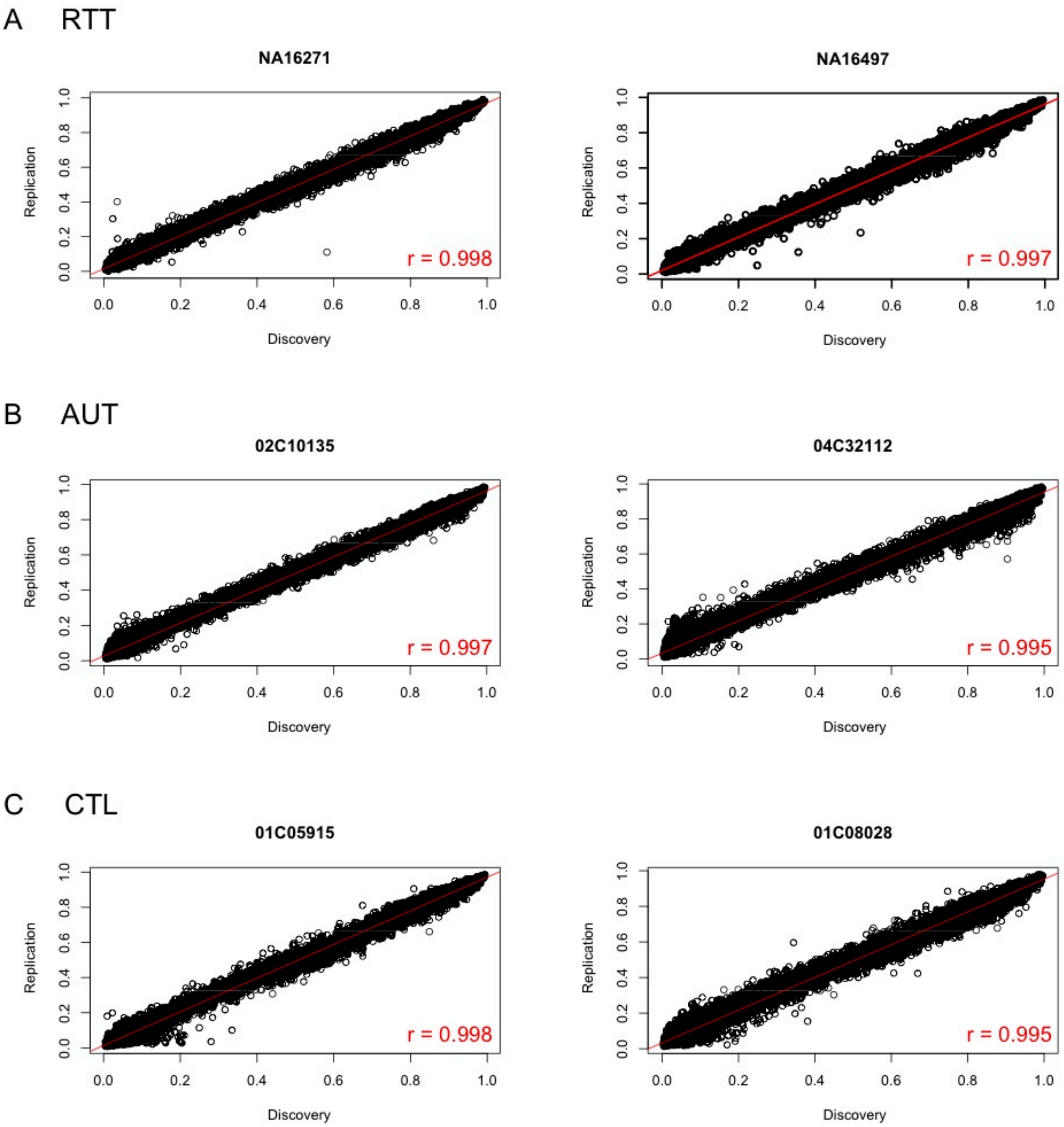

Figure S2. High correlation between technical replicate pairs. Six samples (2 per etiological group) assayed using the Illumina HumanMethylation27 assay in discovery were assayed again in replication. Nearly perfect agreement was observed between all technical replicate pairs (Pearson's r > 0.99). One sample (02C10135) was not included in further analyses.

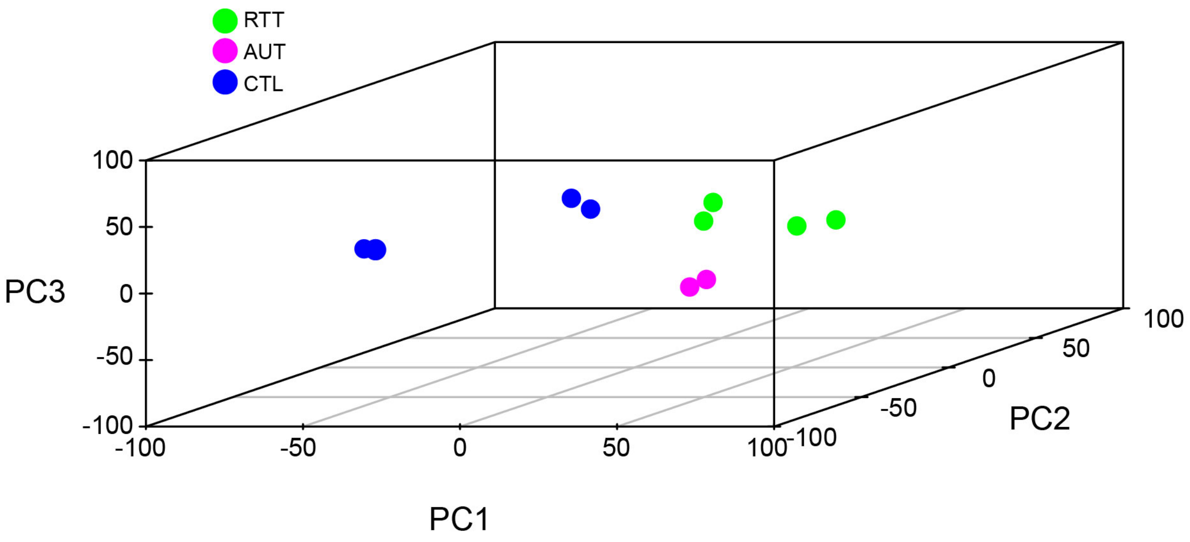

Figure S3. PCA of DNA methylation data from 5 technical replicate pairs. PCA of the 40 probe methylation profile for the 5 replication samples shows tight clustering between replicate pairs. Three principle components (PC1-3), representing 44.0, 30.8, and 15.1% of the variance, respectively, are shown.

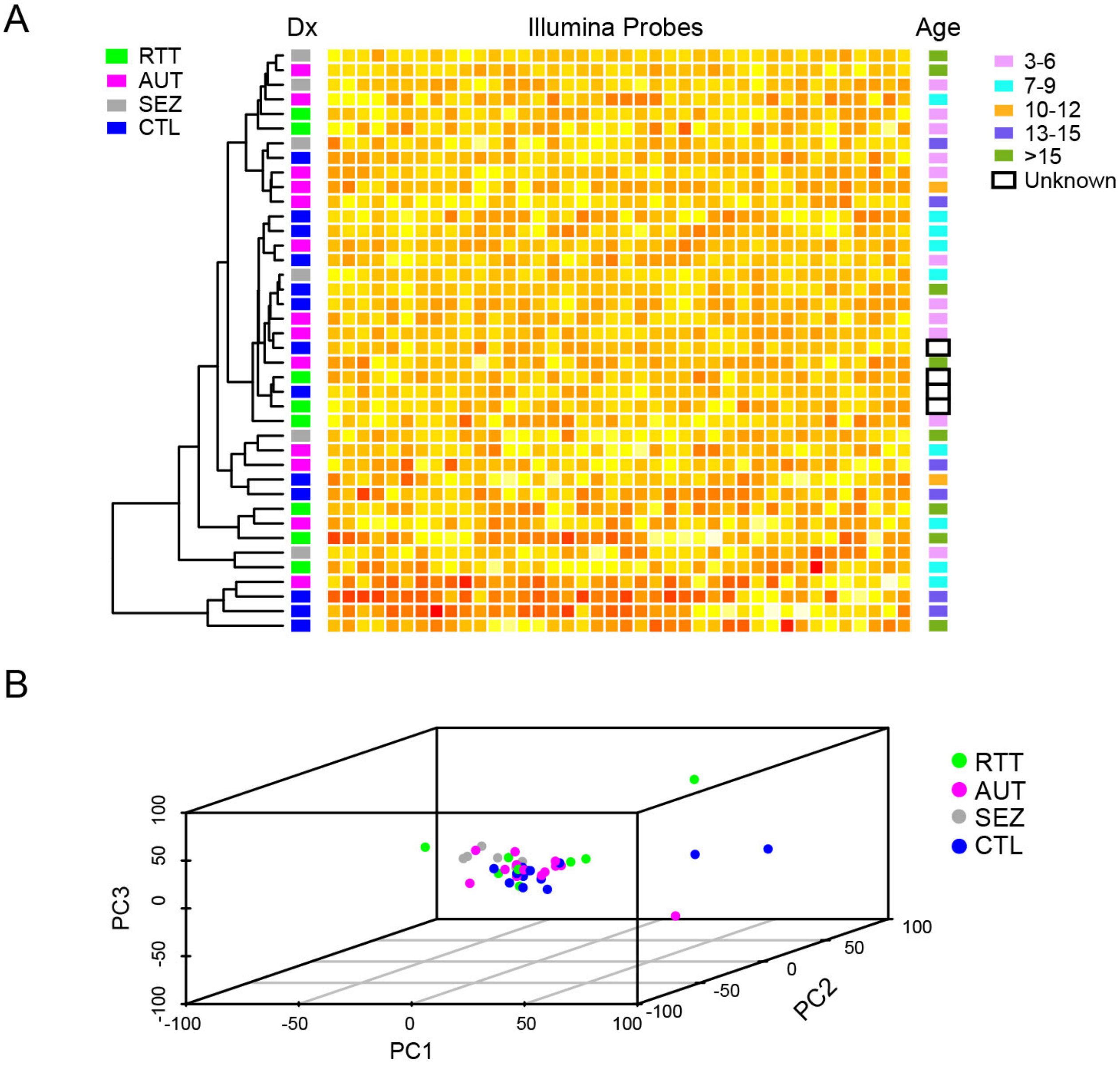

Figure S4. Hierarchical clustering and PCA of DNA methylation data from the replication cohort. The selected 40 probes comparing LCL samples from females diagnosed with neurodevelopmental disorders (RTT, AUT, and SEZ) to CTL females were used for hierarchical clustering and PCA. (A) Hierarchical clustering of the 40 females in the replication cohort. There was no strong clustering of DNA methylation patterns by etiology (or by age). (B) PCA of the 40 probe methylation profile for the 40 replication samples. Three principle components (PC1-3), representing 18.3, 11.6, and 8.0% of the variance, respectively, are shown.

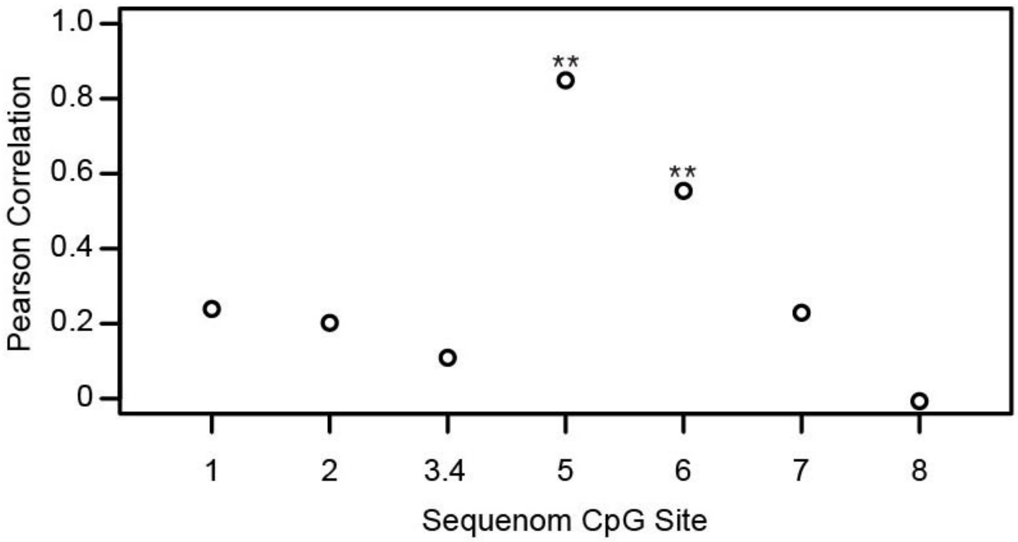

Figure S5. Sequenom DNA methylation validation at the *TAC1* promoter. The *TAC1* Sequenom target region includes 8 CpG sites, 2 of which are overlapping (3 and 4). Mean methylation levels at CpG sites 5 and 6 are significantly correlated with Illumina probe mean methylation levels (*P*<0.00001).

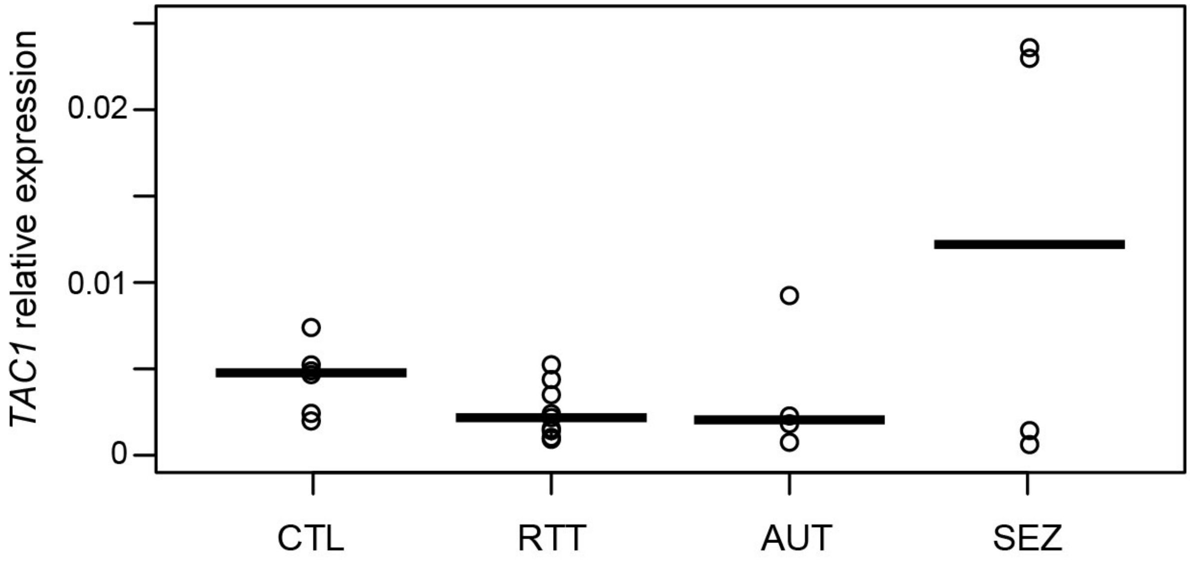

Figure S6. ***TAC1*** **expression in LCLs.** *TAC1* mRNA assayed by qRT-PCR. No significant difference in TAC1 expression was detected between clinical groups. Overall, *TAC1* is weakly expressed in LCLs, consistent with previously reported expression in lymphocytes. Data are presented as mean ± s.e.m.

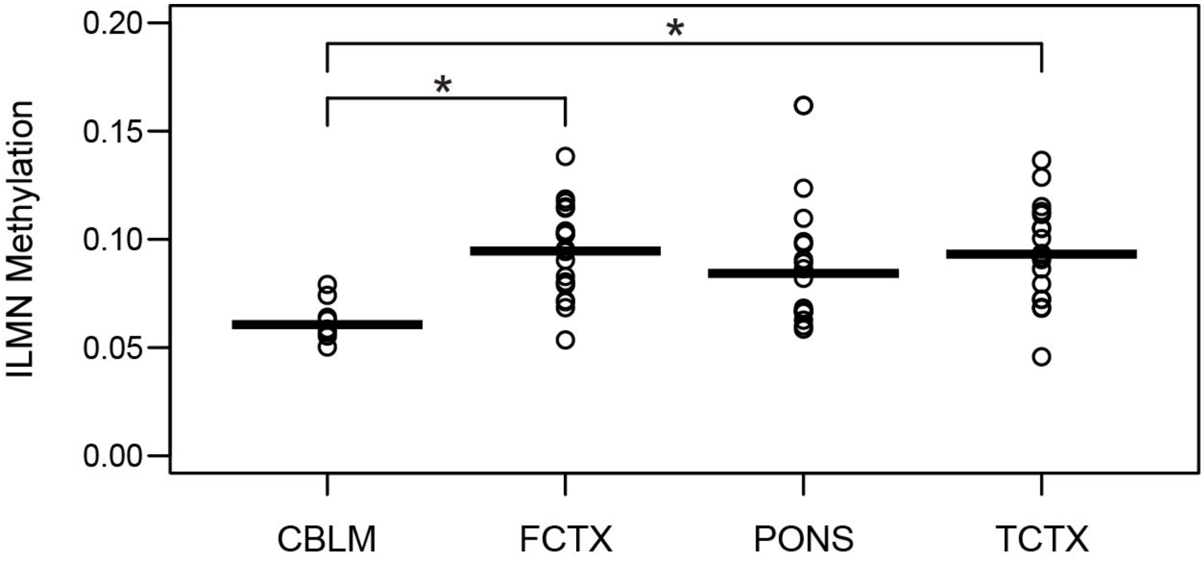

Figure S7. *TAC1* promoter CpG DNA methylation in female postmortem brain. *TAC1* DNA methylation (cg14221171) assayed in cerebellum (CBLM), frontal cortex (FCTX), pons and temporal cortex (TCTX) from control female postmortem brain using the Illumina HumanMethyl27 BeadChip. Significant differential DNA methylation of *TAC1* occurs among distinct brain regions (ANOVA F(3,63)=4.39, *P*=0.007). *TAC1* was significantly hypermethylated in the CBLM relative to TCTX (Tukey *post hoc*, *P*=0.009) and FCTX (*P*=0.006), though there was no differential DNA methylation between the two cerebral cortex regions included in this study (*P*>0.05). **P*>0.01.

Table S1. LCL sample characteristics.

SAMPLE	SOURCE	DX	NUCLEOTIDE	PROTEIN	SEX	AGE	RACE	ILMN	SEQ	LCL
00C04563	RUCDR	AUT			F	23	Euro	2	+	
01C06480	RUCDR	AUT			F	7	Euro	2	+	
01C06497	RUCDR	AUT			F	13	Euro	2	+	
01C07488	RUCDR	AUT			F	9	Euro	2	+	
02C10135	RUCDR	AUT								
02C10223	RUCDR	AUT								
03C15793	RUCDR	AUT			F	12	Euro	2	+	
03C15943	RUCDR	AUT			F	3	Euro	2	+	
03C16444	RUCDR	AUT			F	5	Euro	2	+	+
03C16562	RUCDR	AUT			F	7	Euro	2	+	+
03C17196	RUCDR	AUT			F	15	Euro	2	+	
04C24298	RUCDR	AUT			F	29	Euro	2	+	
04C32112	RUCDR	AUT			F	3	Euro	1,2	+	+
04C35560	RUCDR	AUT			F	6	Euro	1	+	+
04C36042	RUCDR	AUT			F	6	Euro	2	+	
04C36352	RUCDR	AUT			F	15	Euro	1	+	
05C40546	RUCDR	AUT			F	9	Euro	1		
05C42143	RUCDR	AUT			F	14	Euro	2	+	
05C47170	RUCDR	AUT			F	9	Euro	2	+	+
01C05476	RUCDR	CTL			F	14	Euro	1	+	
01C05712	RUCDR	CTL			F	14	Euro	2	+	
01C05898	RUCDR	CTL			F	26	Euro	2	+	
01C05915	RUCDR	CTL			F	3	Euro	1,2	+	+
01C06052	RUCDR	CTL			F	11	Euro	2	+	
01C06061	RUCDR	CTL			F	16	Euro	2	+	
01C06493	RUCDR	CTL			F	7	Euro	2	+	+
01C07313	RUCDR	CTL			F	6	Euro	1	+	+
01C07372	RUCDR	CTL			F	9	Euro	1	+	+
01C08028	RUCDR	CTL			F	13	Euro	1,2	+	+
01C08060	RUCDR	CTL			F	14	Euro	2	+	+
01C08448	RUCDR	CTL			F	15	Euro	1	+	
04C31982	RUCDR	CTL			F	4	Euro	2	+	
04C35584	RUCDR	CTL			F	6	Euro	2	+	
04C36913	RUCDR	CTL			F	5	Euro	2	+	+
05C40435	RUCDR	CTL			F		Euro	2	+	
07C64541	RUCDR	CTL			F	9	Euro	2	+	
07C64722	RUCDR	CTL			F	15	Euro	2	+	
07C68500	RUCDR	CTL			F		Euro	2	+	
ND02110	CCR	EPI			F	9	Euro	2	+	+
ND04353	CCR	EPI			F	3	Euro	2	+	+
ND06755	CCR	EPI			F	30	Euro	2	+	
ND08877	CCR	EPI			F	14	Euro	2	+	+
ND09027	CCR	EPI			F	25	Euro	2	+	
ND21676	CCR	EPI			F	6	Euro	2	+	+
GM07983	CCR	RTT	705delG	E235fs	F	25	Euro	2	+	+

GM11300	CCR	RTT	316C>T	R106W	F			1	+	
GM11301	CCR	RTT	705delG	E235fs	F			2	+	+
GM11304	CCR	RTT	880C>T	R294X	F			1	+	
GM11310	CCR	RTT	502C>T	R168X	F			1	+	+
GM11313	CCR	RTT			F			2		
GM16270	CCR	RTT	763C>T	R255X	F	6	Euro	1	+	+
GM16271	CCR	RTT	473C>T	T158M	F	3	Euro	1,2	+	+
GM16368	CCR	RTT	473C>T	T158M	F	4	Euro	1	+	
GM16382	CCR	RTT	1160_1185del26	P387fs	F	6	Euro	2	+	
GM16405	CCR	RTT	481G>A	G161V	F	6	Euro	2	+	+
GM16480	CCR	RTT	916C>T	R306C	F	14	Euro	1	+	+
GM16497	CCR	RTT	763C>T	R255X	F	13	Euro	1,2	+	+
GM16546	CCR	RTT	880C>T	R294X	F	15	Euro	1	+	
GM16547	CCR	RTT	730C>T	Q244X	F	5	Euro	2	+	+
GM16560	CCR	RTT			F	30	Euro	2		
GM17540	CCR	RTT	401C>G	S134C	F	9	Euro	1	+	+
GM17583	CCR	RTT	298C>G	L100V	F	7	Euro	2		

MECP2 nucleotide and protein mutations are listed where applicable; No mutation is reported in CCR for GM11313 or GM16560; GM17583 is also 46,XXX. Samples included in ILMN discovery (1) or replication (2). Abbreviations: DX, diagnosis; AUT, autism; CCR, Coriell Cell Repositories; CTL, control; Euro, European; RTT, Rett syndrome; RUCDR, Rutgers University Cell and DNA Repository; ILMN, Illumina HumanMethylation27 Assay; SEQ, Sequenom EpiTYPER Assays; LCL, lymphoblastoid cell line.

Table S2. ANOVA results for 391 probes with Δβ≥0.15 in the discovery cohort

			Δβ				
PROBE	GENE	CHR	RTT-CTL	RTT-AUT	AUT-CTL	P	FDR
cg17950095	*HOXA11*	7	-0.13	0.05	-0.18	0.0002	0.03
cg00187686	*TCN1*	11	0.20	-0.02	0.23	0.0002	0.03
cg04527989	*PTCD2*	5	0.00	0.19	-0.18	0.0003	0.03
cg14221171	*TAC1*	7	0.07	-0.18	0.25	0.0004	0.03
cg03718539	*ETNK2*	1	-0.08	-0.18	0.10	0.0004	0.03
cg09242541	*APITD1*	1	0.29	0.08	0.21	0.0006	0.04
cg18432105	*MYH2*	17	0.16	0.12	0.04	0.001	0.06
cg14986136	*WBP5*	X	-0.19	0.01	-0.19	0.001	0.06
cg18838701	*TNNI3*	19	0.06	-0.21	0.27	0.002	0.06
cg06537230	*DLX5*	7	0.13	-0.06	0.19	0.002	0.06
cg19002579	*SMPX*	X	0.22	-0.05	0.27	0.002	0.06
cg06618866	*TLR2*	4	0.07	-0.10	0.17	0.002	0.06
cg23196831	*COL14A1*	8	0.13	-0.03	0.16	0.002	0.06
cg01541443	*C7orf41*	7	-0.01	0.17	-0.18	0.002	0.06

cg19642007	*TNNT3*	11	0.14	0.17	-0.02	0.003	0.06
cg00176210	*ANK1*	8	0.15	-0.05	0.21	0.003	0.06
cg02049180	*INSRR*	1	0.01	0.22	-0.21	0.003	0.06
cg09868035	*C20orf135*	20	0.16	0.07	0.09	0.003	0.07
cg26227465	*IFNG*	12	0.21	-0.05	0.25	0.003	0.07
cg20322862	*TGIF1*	18	-0.04	0.14	-0.19	0.003	0.07
cg09404633	*LMOD1*	1	0.19	-0.03	0.22	0.004	0.07
cg04555771	*CACNA2D2*	3	0.17	-0.11	0.28	0.004	0.07
cg10467098	*C11orf68*	11	-0.06	0.14	-0.21	0.005	0.08
cg04091078	*SLCO1C1*	12	0.02	0.15	-0.13	0.01	0.09
cg05570980	*C3orf52*	3	0.05	-0.13	0.17	0.01	0.09
cg02441647	*COL8A1*	3	-0.17	-0.05	-0.12	0.01	0.09
cg18801691	*DCC*	18	-0.12	0.05	-0.17	0.01	0.09
cg21306775	*FLJ44881*	18	0.21	0.15	0.06	0.01	0.09
cg14141399	*HAS1*	19	0.20	0.11	0.10	0.01	0.09
cg12815142	*SPAG7*	17	0.16	0.13	0.03	0.01	0.09
cg14062083	*KRTAP13-4*	21	0.16	0.14	0.02	0.01	0.09
cg20311730	*NLRP10*	11	0.17	0.04	0.13	0.01	0.09
cg00415993	*F2RL2*	5	0.23	0.02	0.21	0.01	0.09
cg00318573	*CHRNA4*	20	0.18	0.14	0.04	0.01	0.09
cg15350036	*CROT*	7	0.16	0.07	0.09	0.01	0.09
cg04993257	*PLAC2*	19	0.18	0.10	0.08	0.01	0.09
cg03273615	*RBM41*	X	0.18	0.07	0.11	0.01	0.09
cg11505048	*APOBEC4*	1	-0.05	0.11	-0.16	0.01	0.09
cg01145396	*CHRNG*	2	0.16	0.01	0.15	0.01	0.09
cg13370916	*STARD8*	X	0.18	0.06	0.12	0.01	0.09
cg24432916	*SOX3*	X	0.16	0.02	0.14	0.01	0.10
cg18420965	*EPHA5*	4	0.06	-0.16	0.23	0.01	0.10
cg08109815	*NMBR*	6	0.11	-0.15	0.26	0.01	0.10
cg02244695	*TMEM176A*	7	0.06	-0.13	0.19	0.01	0.10
cg21958798	*GUCY1A2*	11	0.00	-0.19	0.19	0.01	0.10
cg16176600	*FRK*	6	-0.04	0.21	-0.24	0.01	0.10
cg00179446	*UPF3B*	X	0.18	0.01	0.17	0.01	0.10
cg22158923	*C7orf52*	7	0.16	0.18	-0.02	0.01	0.10
cg26135325	*LCE3A*	1	0.20	0.11	0.09	0.01	0.10
cg17582777	*EFNA3*	1	0.05	0.22	-0.17	0.01	0.10
cg15988232	*CSPG5*	3	-0.23	-0.15	-0.08	0.01	0.10
cg24693053	*MFSD7*	4	0.03	-0.13	0.16	0.01	0.10
cg10635061	*FHL2*	2	0.16	0.15	0.01	0.01	0.10
cg05467458	*SLC7A9*	19	-0.12	0.09	-0.21	0.01	0.10
cg21400640	*TMSB4X*	X	0.20	0.00	0.20	0.01	0.10
cg00347729	*MMP10*	11	0.06	0.16	-0.11	0.01	0.10
cg03562120	*WISP2*	20	0.09	0.16	-0.07	0.01	0.10
cg00666746	*SYDE1*	19	0.16	0.13	0.03	0.02	0.10
cg12949760	*KCNQ1*	11	0.15	0.16	-0.01	0.02	0.10
cg09040752	*AOC3*	17	0.03	0.16	-0.13	0.02	0.10
cg05535113	*CHST4*	16	0.10	-0.14	0.24	0.02	0.10

cg24986868	*PKP4*	2	0.15	0.03	0.12	0.02	0.10
cg11098259	*AQP9*	15	0.07	0.19	-0.12	0.02	0.10
cg24522076	*FOXO4*	X	0.16	0.04	0.12	0.02	0.10
cg07057831	*STX2*	12	-0.20	-0.08	-0.13	0.02	0.10
cg13995453	*KLRB1*	12	0.05	0.16	-0.11	0.02	0.10
cg04456238	*WT1*	11	-0.20	0.05	-0.26	0.02	0.10
cg01532080	*SSBP4*	19	0.02	-0.13	0.15	0.02	0.10
cg14732540	*BRDT*	1	0.00	0.23	-0.23	0.02	0.10
cg19504888	*MFF*	2	0.23	0.11	0.11	0.02	0.10
cg07156669	*CPD*	17	0.21	0.05	0.16	0.02	0.10
cg01643580	*KCNK3*	2	0.10	-0.12	0.22	0.02	0.10
cg19769182	*PRRT2*	16	0.02	-0.20	0.21	0.02	0.10
cg02679745	*FUT7*	9	0.17	-0.07	0.24	0.02	0.10
cg07749074	*HRG*	3	0.17	0.11	0.06	0.02	0.10
cg05660795	*IGFBP1*	7	0.10	-0.13	0.22	0.02	0.10
cg09339527	*ZFP2*	5	0.07	-0.14	0.21	0.02	0.10
cg22980351	*DCAF12L1*	X	0.16	-0.06	0.22	0.02	0.10
cg03258472	*CRB3*	19	0.08	-0.09	0.17	0.02	0.10
cg22598563	*P4HA2*	5	-0.03	-0.16	0.14	0.02	0.10
cg01309671	*FAM122C*	X	0.12	-0.04	0.16	0.02	0.10
cg22892904	*CBX2*	17	0.09	-0.16	0.25	0.02	0.10
cg02569613	*C10orf72*	10	0.04	-0.15	0.19	0.02	0.10
cg20588045	*PCDH15*	10	0.15	-0.07	0.22	0.02	0.10
cg04675937	*CDKN2B*	9	-0.16	0.01	-0.17	0.02	0.10
cg16977035	*ALX4*	11	0.15	-0.01	0.16	0.02	0.10
cg08241785	*F2RL2*	5	0.15	-0.06	0.21	0.02	0.10
cg03138668	*SLC17A7*	19	-0.07	-0.18	0.11	0.02	0.10
cg23033024	*CST8*	20	0.10	0.16	-0.05	0.02	0.10
cg26320696	*PARVA*	11	0.02	-0.18	0.20	0.02	0.10
cg04975920	*TSPAN1*	1	0.17	-0.02	0.19	0.02	0.10
cg15425811	*C22orf27*	22	0.17	-0.05	0.22	0.03	0.10
cg25148589	*GRIA2*	4	0.16	0.05	0.10	0.03	0.10
cg22971191	*SLC10A2*	13	-0.07	0.15	-0.22	0.03	0.10
cg01112778	*PPP2R2B*	5	0.07	0.18	-0.12	0.03	0.10
cg02983451	*KLF11*	2	0.01	-0.14	0.16	0.03	0.11
cg02978737	*PVALB*	22	0.05	0.15	-0.10	0.03	0.11
cg14611112	*LCN6*	9	0.04	0.17	-0.13	0.03	0.11
cg20908204	*DMPK*	19	0.23	0.13	0.09	0.03	0.11
cg18433380	*NNAT*	20	0.16	0.09	0.06	0.03	0.11
cg06933965	*CMKLR1*	12	0.17	-0.01	0.18	0.03	0.11
cg06851207	*PNMAL1*	19	-0.12	0.16	-0.29	0.03	0.11
cg15787039	*SCG5*	15	0.16	-0.04	0.21	0.03	0.11
cg19945840	*B3GALT6*	1	0.14	-0.08	0.22	0.03	0.11
cg02847500	*SOX3*	X	0.15	-0.02	0.18	0.03	0.11
cg05836145	*SP8*	7	0.13	-0.06	0.19	0.03	0.11
cg09059635	*NTNG2*	9	0.14	-0.07	0.21	0.03	0.11
cg04005707	*CWH43*	4	0.18	0.08	0.10	0.03	0.11

cg21934269	*RPL39*	X	-0.06	0.12	-0.18	0.03	0.11
cg07732644	*ASPA*	17	0.05	0.18	-0.12	0.03	0.11
cg18006568	*ANKRD53*	2	0.09	-0.07	0.16	0.03	0.11
cg14520892	*POLA1*	X	0.15	0.04	0.11	0.03	0.11
cg12135976	*SLC5A8*	12	0.05	-0.11	0.16	0.03	0.11
cg26251865	*IRGC*	19	0.16	0.08	0.08	0.03	0.11
cg18641937	*ITLN2*	1	0.14	-0.07	0.21	0.03	0.11
cg12864235	*CDH9*	5	0.02	0.21	-0.19	0.03	0.11
cg16682903	*ACVR1*	2	0.05	0.15	-0.10	0.03	0.11
cg23704362	*C8orf46*	8	-0.06	0.14	-0.20	0.03	0.11
cg20506783	*ARHGAP22*	10	-0.16	0.05	-0.21	0.03	0.11
cg22224704	*GSTP1*	11	0.19	0.11	0.08	0.04	0.11
cg15819333	*COX4I1*	16	-0.15	-0.12	-0.04	0.04	0.11
cg12434779	*ARHGAP6*	X	0.14	-0.04	0.18	0.04	0.11
cg05706061	*SLC31A2*	9	-0.18	0.06	-0.23	0.04	0.11
cg05389183	*PPIC*	5	-0.17	-0.03	-0.14	0.04	0.11
cg01782486	*ZBTB7B*	1	0.15	0.08	0.07	0.04	0.11
cg01880569	*CDH13*	16	-0.16	0.09	-0.25	0.04	0.11
cg06811800	*ATP4B*	13	0.05	0.18	-0.14	0.04	0.11
cg05260966	*TIMP3*	22	0.17	0.07	0.11	0.04	0.11
cg11320084	*RNF2*	1	0.21	0.04	0.17	0.04	0.11
cg22461835	*ADRA1A*	8	0.07	-0.13	0.20	0.04	0.11
cg04317399	*HOXA4*	7	0.17	-0.04	0.21	0.04	0.11
cg08508325	*CARS*	11	-0.18	-0.10	-0.07	0.04	0.11
cg13488201	*ADAM12*	10	0.10	-0.07	0.17	0.04	0.11
cg24995836	*GABRA3*	X	0.07	-0.09	0.16	0.04	0.11
cg19403023	*TESSP1*	16	0.20	0.04	0.16	0.04	0.11
cg12610070	*TSPAN15*	10	0.09	0.16	-0.07	0.04	0.11
cg25431974	*ECEL1*	2	0.15	0.03	0.13	0.04	0.11
cg12348970	*SLC24A2*	9	0.12	0.17	-0.05	0.04	0.11
cg00344372	*HGFAC*	4	0.12	-0.07	0.19	0.04	0.11
cg13473336	*SLC5A2*	16	0.02	0.18	-0.16	0.04	0.11
cg01851399	*PKP2*	12	0.01	-0.16	0.17	0.04	0.11
cg14338887	*GNMT*	6	-0.03	-0.19	0.15	0.04	0.11
cg13269407	*C22orf26*	22	0.25	0.22	0.03	0.04	0.11
cg17861230	*PDE4C*	19	0.19	0.07	0.12	0.04	0.11
cg08927738	*BCAS1*	20	0.21	0.10	0.10	0.04	0.12
cg04037732	*NLGN3*	X	0.19	0.08	0.12	0.04	0.12
cg09523691	*ATG12*	5	-0.02	0.14	-0.16	0.04	0.12
cg13883681	*GOT2*	16	-0.04	0.14	-0.19	0.04	0.12
cg05055150	*MAG*	19	-0.16	0.00	-0.16	0.04	0.12
cg06454226	*METTL4*	18	0.21	0.06	0.16	0.04	0.12
cg16022279	*ZNF185*	X	-0.01	0.15	-0.16	0.04	0.12
cg01813965	*CCDC135*	16	0.17	0.13	0.04	0.05	0.12
cg07705908	*SPDEF*	6	0.04	0.16	-0.12	0.05	0.12
cg11619390	*ZBTB8A*	1	0.18	0.02	0.15	0.05	0.12
cg16935609	*SRD5A1*	5	-0.09	0.09	-0.18	0.05	0.12

cg11115702	*SPNS1*	16	-0.04	-0.17	0.14	0.05	0.12
cg06750167	*TESC*	12	0.18	0.02	0.16	0.05	0.12
cg20781967	*NINJ2*	12	0.07	-0.14	0.21	0.05	0.13
cg01526089	*P2RX1*	17	0.24	-0.04	0.27	0.05	0.13
cg10660256	*BHMT*	5	0.02	-0.16	0.18	0.05	0.13
cg17095731	*LRP8*	1	0.23	0.08	0.15	0.05	0.13
cg14724265	*PPEF2*	4	0.15	-0.03	0.18	0.05	0.13
cg08935301	*LMCD1*	3	-0.09	0.13	-0.21	0.05	0.13
cg08263647	*LXN*	3	-0.05	-0.17	0.12	0.05	0.13
cg21856603	*ITGAE*	17	0.18	-0.05	0.22	0.05	0.13
cg19668234	*PRSS50*	3	0.17	-0.06	0.23	0.05	0.13
cg10161121	*FASLG*	1	0.02	0.16	-0.14	0.05	0.13
cg20774846	*DPYS*	8	0.12	0.16	-0.04	0.05	0.13
cg04058169	*BUB3*	10	0.17	0.09	0.08	0.05	0.13
cg26354398	*RGS3*	9	0.06	0.16	-0.11	0.06	0.13
cg10670077	*INSR*	19	0.15	0.00	0.16	0.06	0.13
cg09280976	*MEG3*	14	-0.05	0.11	-0.16	0.06	0.13
cg03918304	*HOXD10*	2	0.17	0.01	0.16	0.06	0.13
cg21410991	*ISL1*	5	0.14	-0.06	0.20	0.06	0.13
cg20792062	*KCNA5*	12	0.01	-0.14	0.16	0.06	0.13
cg01805282	*EYA4*	6	-0.01	-0.20	0.19	0.06	0.13
cg08586737	*GCC1*	7	0.13	-0.07	0.20	0.06	0.13
cg18946226	*MYO16*	13	-0.13	0.07	-0.20	0.06	0.13
cg12638745	*LCA5L*	21	0.17	0.12	0.05	0.06	0.13
cg02218324	*RSPH6A*	19	0.19	0.07	0.12	0.06	0.13
cg20798152	*CARTPT*	5	-0.10	0.08	-0.18	0.06	0.13
cg20469837	*GALNT5*	2	0.17	-0.01	0.19	0.06	0.13
cg26394940	*C22orf26*	22	0.22	0.17	0.05	0.06	0.13
cg09848074	*KLRG2*	7	0.13	0.19	-0.06	0.06	0.13
cg13749822	*HHIP*	4	0.11	0.19	-0.08	0.06	0.13
cg26581729	*NPDC1*	9	-0.08	0.16	-0.24	0.06	0.13
cg09816471	*SNN*	16	0.12	-0.17	0.29	0.06	0.13
cg23545272	*EFNB1*	X	0.15	0.09	0.07	0.06	0.13
cg24132694	*SNAPC2*	19	-0.19	0.06	-0.25	0.06	0.13
cg18743730	*CA4*	17	0.14	-0.02	0.16	0.07	0.13
cg00708598	*LECT1*	13	0.05	-0.10	0.16	0.07	0.13
cg12108912	*TMEM177*	2	0.00	0.16	-0.16	0.07	0.13
cg15096140	*MYO1B*	2	-0.18	0.09	-0.27	0.07	0.13
cg02131995	*HAMP*	19	0.14	-0.08	0.22	0.07	0.13
cg22016649	*PNPLA2*	11	-0.12	0.10	-0.22	0.07	0.13
cg02888247	*C6orf62*	6	-0.10	0.06	-0.16	0.07	0.14
cg17067005	*IL10*	1	0.19	-0.02	0.21	0.07	0.14
cg21109025	*CCL2*	17	0.03	0.19	-0.16	0.07	0.14
cg09947274	*CAD*	2	0.06	0.15	-0.09	0.07	0.14
cg24477567	*EPOR*	19	0.21	0.03	0.18	0.07	0.14
cg12619509	*DNASE1L2*	16	0.06	-0.15	0.20	0.07	0.14
cg25978208	*SNRPN*	15	0.10	-0.07	0.17	0.07	0.14

cg03330058	*ABTB1*	3	0.20	0.11	0.10	0.07	0.14
cg20857253	*TCP10L*	21	-0.14	-0.20	0.05	0.07	0.14
cg10756887	*C22orf15*	22	0.03	-0.15	0.18	0.07	0.14
cg06657741	*SLC9A6*	X	-0.08	0.08	-0.17	0.07	0.14
cg16046465	*FRMD5*	15	-0.01	-0.16	0.15	0.07	0.14
cg19859270	*GPR15*	3	0.21	0.08	0.13	0.07	0.14
cg16632715	*HOXD11*	2	0.07	-0.08	0.15	0.08	0.14
cg24429836	*LDHD*	16	0.07	0.16	-0.09	0.08	0.14
cg07103493	*SLC27A6*	5	0.03	-0.15	0.18	0.08	0.14
cg08416046	*IRS4*	X	0.13	-0.04	0.16	0.08	0.14
cg15539420	*HOXB8*	17	0.08	-0.12	0.20	0.08	0.14
cg23855093	*GPR128*	3	-0.11	0.08	-0.19	0.08	0.14
cg06710648	*DAB1*	1	-0.04	-0.18	0.14	0.08	0.14
cg03387723	*SCMH1*	1	-0.20	-0.09	-0.11	0.08	0.14
cg22870280	*BCL2L10*	15	0.09	0.18	-0.08	0.08	0.14
cg09924998	*VPS33A*	12	-0.09	0.11	-0.21	0.08	0.14
cg18727700	*SRPX2*	X	0.15	0.11	0.04	0.08	0.15
cg01988129	*ADHFE1*	8	0.05	-0.16	0.21	0.08	0.15
cg00846036	*DLG2*	11	0.12	0.20	-0.08	0.08	0.15
cg09573795	*MSX1*	4	0.12	-0.07	0.19	0.08	0.15
cg19055639	*ARMCX2*	X	0.14	-0.01	0.15	0.08	0.15
cg00278366	*RAD9B*	12	-0.06	0.10	-0.16	0.09	0.15
cg22830895	*CRYGN*	7	0.04	0.15	-0.11	0.09	0.15
cg19384697	*UPK3B*	7	0.13	0.15	-0.02	0.09	0.15
cg06263495	*ASCL2*	11	0.05	-0.11	0.16	0.09	0.15
cg01593385	*FGG*	4	0.04	0.16	-0.12	0.09	0.15
cg25201363	*LRPAP1*	4	0.16	0.08	0.08	0.09	0.15
cg22375192	*IGF1R*	15	0.02	-0.14	0.16	0.09	0.15
cg04513422	*C13orf29*	13	0.10	-0.12	0.22	0.09	0.15
cg04123409	*SDS*	12	0.08	-0.08	0.16	0.09	0.15
cg03534410	*TMEM40*	3	0.16	0.06	0.10	0.09	0.15
cg20831708	*SEC31B*	10	0.04	-0.17	0.21	0.09	0.15
cg03743584	*PRAP1*	10	0.13	-0.05	0.19	0.09	0.15
cg15262984	*UBE2D4*	7	0.02	-0.14	0.15	0.10	0.16
cg15422147	*SERPINB5*	18	-0.04	0.14	-0.17	0.10	0.16
cg15146752	*EPHA2*	1	-0.11	0.09	-0.20	0.10	0.16
cg16449464	*MAP1D*	2	-0.03	0.16	-0.19	0.10	0.16
cg18849169	*GPX3*	5	-0.08	0.08	-0.16	0.10	0.16
cg20092036	*UBE3B*	12	0.16	0.02	0.14	0.10	0.16
cg22035229	*MSH4*	1	-0.02	0.13	-0.15	0.10	0.16
cg15383120	*DUSP22*	6	0.09	-0.14	0.24	0.10	0.16
cg24447042	*SMARCA1*	X	0.11	-0.04	0.15	0.10	0.16
cg26609631	*GSX1*	13	0.09	-0.07	0.15	0.10	0.16
cg20269537	*ATXN10*	22	-0.09	0.10	-0.18	0.10	0.16
cg17421623	*KTELC1*	3	-0.04	0.15	-0.20	0.10	0.16
cg07959477	*CLEC1A*	12	-0.07	0.11	-0.17	0.10	0.16
cg14550066	*NCR1*	19	0.16	0.01	0.15	0.10	0.16

cg19815720	*HTR3C*	3	-0.08	0.08	-0.16	0.10	0.16
cg00615377	*RBM9*	22	0.06	-0.11	0.17	0.10	0.16
cg25344672	*C9orf171*	9	0.15	-0.06	0.21	0.10	0.16
cg06675478	*SOX1*	13	0.11	-0.04	0.15	0.11	0.16
cg16051685	*TRIM63*	1	0.14	-0.02	0.15	0.11	0.16
cg08145625	*TRAM1L1*	4	0.05	-0.13	0.18	0.11	0.16
cg02735486	*ANK2*	4	0.08	-0.11	0.19	0.11	0.16
cg20723355	*FBXO39*	17	0.16	0.01	0.15	0.11	0.16
cg23065097	*FKBP1B*	2	0.02	0.18	-0.16	0.11	0.16
cg06432655	*CLIP3*	19	0.15	0.03	0.12	0.11	0.17
cg18536148	*TBX4*	17	-0.06	-0.16	0.10	0.11	0.17
cg17408686	*CHCHD6*	3	0.06	-0.13	0.19	0.11	0.17
cg22799132	*TRPC5*	X	0.09	-0.10	0.19	0.11	0.17
cg10076009	*SMARCA1*	X	-0.15	-0.03	-0.13	0.11	0.17
cg20648149	*SYNE2*	14	-0.07	0.10	-0.17	0.11	0.17
cg23771929	*FREQ*	9	-0.16	-0.07	-0.08	0.11	0.17
cg10189695	*GPR78*	4	0.07	-0.15	0.21	0.11	0.17
cg20587168	*ACTL9*	19	0.16	0.09	0.07	0.11	0.17
cg13842648	*IL9*	5	-0.04	0.16	-0.20	0.12	0.17
cg24710073	*TBP*	6	0.00	0.17	-0.18	0.12	0.17
cg24516901	*FAM124B*	2	0.04	-0.11	0.16	0.12	0.17
cg10608341	*HESX1*	3	0.05	0.18	-0.14	0.12	0.17
cg04995717	*TEK*	9	0.16	0.09	0.07	0.12	0.17
cg22016571	*RP6-213H19.1*	X	0.06	-0.09	0.15	0.12	0.17
cg18438777	*NPY5R*	4	0.11	-0.04	0.15	0.12	0.17
cg09893305	*HAPLN1*	5	-0.20	-0.03	-0.18	0.12	0.17
cg03330678	*SEPT9*	17	-0.11	0.08	-0.19	0.12	0.17
cg00418150	*ALX4*	11	0.06	-0.10	0.16	0.12	0.17
cg08812936	*KCNMB2*	3	0.14	-0.04	0.18	0.13	0.18
cg22189019	*FGF22*	19	0.20	0.04	0.15	0.13	0.18
cg13950558	*BLOC1S3*	19	-0.11	0.05	-0.17	0.13	0.18
cg13633026	*MS4A8B*	11	0.10	-0.07	0.16	0.13	0.18
cg15684563	*TSPAN8*	12	0.18	0.06	0.13	0.13	0.18
cg12473775	*RHOD*	11	0.13	0.19	-0.06	0.13	0.18
cg26201213	*MGMT*	10	-0.11	0.05	-0.16	0.13	0.18
cg24854010	*WNT5B*	12	-0.04	0.16	-0.20	0.13	0.18
cg22678136	*SNRPN*	15	0.18	0.04	0.14	0.13	0.18
cg14944269	*RAB15*	14	-0.18	-0.04	-0.14	0.14	0.18
cg16615211	*TMEM42*	3	0.02	0.17	-0.15	0.14	0.18
cg23878206	*ATF5*	19	-0.05	0.10	-0.15	0.14	0.18
cg14473924	*PDZRN3*	3	0.12	-0.03	0.16	0.14	0.19
cg11822932	*LMO2*	11	0.18	-0.03	0.21	0.14	0.19
cg18752854	*TNS1*	2	-0.02	0.13	-0.15	0.14	0.19
cg14861570	*MMD*	17	0.01	0.16	-0.15	0.14	0.19
cg15720535	*AGPAT2*	9	-0.14	0.06	-0.20	0.14	0.19
cg04001333	*FLVCR2*	14	0.17	-0.02	0.19	0.14	0.19
cg13530039	*CHRM1*	11	0.03	0.16	-0.13	0.14	0.19

cg09245073	*ASGR1*	17	0.15	0.10	0.06	0.14	0.19
cg15284635	*SLC6A11*	3	0.07	-0.10	0.17	0.14	0.19
cg16842214	*KBTBD5*	3	-0.07	0.09	-0.16	0.15	0.19
cg03382797	*C9orf98*	9	0.13	-0.03	0.16	0.15	0.19
cg24585690	*IL9*	5	-0.04	0.16	-0.20	0.15	0.19
cg06048973	*ACTC1*	15	0.13	0.17	-0.05	0.15	0.19
cg12006284	*WT1*	11	-0.06	0.15	-0.21	0.15	0.20
cg02085507	*TRIP10*	19	-0.02	0.14	-0.17	0.15	0.20
cg07285276	*RAPGEF1*	9	-0.01	0.18	-0.19	0.15	0.20
cg02628202	*LGMN*	14	-0.15	-0.05	-0.10	0.15	0.20
cg13446199	*PSCA*	8	0.05	0.18	-0.13	0.16	0.20
cg22815110	*FOXD3*	1	0.06	-0.10	0.16	0.16	0.20
cg25979644	*ATP8B1*	18	-0.06	0.13	-0.19	0.16	0.20
cg01154537	*C10orf4*	10	-0.01	-0.16	0.15	0.16	0.20
cg23741330	*FAM26E*	6	0.12	-0.03	0.16	0.16	0.20
cg14992108	*SNTB1*	8	-0.07	0.08	-0.15	0.16	0.20
cg01946401	*RUNX2*	6	0.06	0.16	-0.10	0.16	0.20
cg25697314	*IL26*	12	-0.08	0.09	-0.17	0.16	0.21
cg21126707	*MYF5*	12	0.12	-0.04	0.16	0.17	0.21
cg02417408	*PPM1M*	3	-0.10	0.06	-0.16	0.17	0.21
cg03544320	*CRMP1*	4	0.20	0.04	0.16	0.17	0.21
cg20322977	*CYP26C1*	10	0.14	-0.01	0.15	0.17	0.21
cg19868730	*POPDC2*	3	0.08	-0.11	0.19	0.17	0.21
cg00816620	*C1orf25*	1	0.14	-0.05	0.18	0.17	0.21
cg24389359	*CCDC63*	12	-0.15	-0.15	0.00	0.17	0.21
cg07054641	*WDR52*	3	0.09	-0.11	0.20	0.17	0.21
cg10282491	*CLDN9*	16	-0.09	0.09	-0.18	0.18	0.21
cg21256649	*PRKAR1A*	17	-0.16	-0.04	-0.12	0.18	0.22
cg09081544	*MUC13*	3	0.13	-0.06	0.19	0.18	0.22
cg01888601	*AGBL2*	11	0.02	0.17	-0.14	0.18	0.22
cg07688613	*INTS3*	1	-0.13	0.03	-0.16	0.18	0.22
cg00202711	*HTR2C*	X	-0.14	0.01	-0.16	0.18	0.22
cg08558340	*SRRT*	7	-0.08	0.08	-0.16	0.19	0.22
cg12467090	*PIK3C2B*	1	0.11	-0.05	0.16	0.19	0.22
cg16080552	*TACSTD2*	1	-0.15	0.02	-0.17	0.19	0.23
cg00698688	*SULT2B1*	19	0.00	0.15	-0.15	0.19	0.23
cg24194775	*NPR2*	9	0.01	-0.15	0.16	0.19	0.23
cg17166338	*TERT*	5	0.04	0.18	-0.14	0.19	0.23
cg02498063	*CCDC60*	12	0.09	-0.06	0.15	0.20	0.23
cg01870826	*LOC389458*	7	0.11	-0.06	0.17	0.20	0.23
cg11428724	*PAX7*	1	0.10	-0.06	0.16	0.20	0.23
cg24652919	*THOC6*	16	0.16	-0.01	0.17	0.20	0.23
cg12762799	*CLEC4E*	12	0.09	-0.07	0.17	0.20	0.23
cg15989091	*LOXL3*	2	0.15	-0.05	0.20	0.21	0.24
cg01757745	*C10orf93*	10	0.13	-0.04	0.18	0.21	0.24
cg21742836	*PPP4C*	16	-0.13	0.06	-0.19	0.22	0.25
cg21418052	*B3GALTL*	13	-0.13	0.05	-0.17	0.22	0.25

cg17387870	*CHFR*	12	0.01	0.18	-0.16	0.22	0.25
cg24471894	*KIAA0020*	9	0.15	0.09	0.06	0.22	0.25
cg02250594	*ONECUT2*	18	0.10	-0.05	0.16	0.22	0.25
cg24211388	*AIF1*	6	0.07	-0.11	0.18	0.23	0.25
cg11235426	*DUSP22*	6	0.13	-0.09	0.22	0.23	0.26
cg22778981	*ERBB2*	17	0.12	-0.10	0.22	0.23	0.26
cg10281770	*WDR45L*	17	-0.03	0.14	-0.18	0.23	0.26
cg00242839	*RBM17*	10	-0.01	0.16	-0.16	0.24	0.26
cg19007731	*RUNX1T1*	8	0.13	-0.03	0.16	0.24	0.26
cg01386493	*ADRB3*	8	-0.09	0.06	-0.16	0.24	0.27
cg08861115	*IL1F9*	2	-0.03	0.15	-0.18	0.24	0.27
cg12552392	*NFS1*	20	-0.18	-0.08	-0.10	0.24	0.27
cg23418591	*APCDD1L*	20	-0.07	0.09	-0.16	0.25	0.27
cg26279025	*IL11*	19	-0.06	0.09	-0.15	0.25	0.27
cg09548084	*SLC35B3*	6	0.17	0.03	0.14	0.25	0.27
cg22282672	*ZNF474*	5	0.09	0.16	-0.07	0.26	0.28
cg06268694	*CELSR1*	22	0.07	-0.11	0.18	0.26	0.28
cg13745870	*SPATA12*	3	0.15	0.05	0.10	0.26	0.28
cg18979223	*CDKN2B*	9	-0.11	0.06	-0.17	0.26	0.28
cg07314984	*PHKA2*	X	-0.10	0.05	-0.15	0.26	0.28
cg12936220	*RPL26L1*	5	-0.08	0.09	-0.17	0.27	0.29
cg14483391	*HTR3D*	3	0.06	-0.10	0.17	0.28	0.29
cg07974891	*ITGB1BP1*	2	0.08	-0.07	0.15	0.28	0.29
cg25003924	*ARPP19*	15	-0.11	0.05	-0.16	0.28	0.29
cg02863947	*NR1I2*	3	0.13	-0.03	0.16	0.29	0.31
cg03439703	*PDE3B*	11	0.16	0.09	0.07	0.29	0.31
cg17003970	*CHFR*	12	0.12	0.17	-0.05	0.29	0.31
cg04645174	*OR7A17*	19	-0.11	0.04	-0.15	0.30	0.32
cg11052143	*ALS2CR11*	2	0.05	-0.11	0.16	0.30	0.32
cg22747092	*NACC2*	9	0.09	-0.08	0.17	0.30	0.32
cg10978355	*CKMT2*	5	-0.09	-0.16	0.07	0.31	0.33
cg17264618	*ENTPD3*	3	0.07	-0.09	0.16	0.31	0.33
cg08126211	*KAAG1*	6	0.09	-0.12	0.22	0.32	0.33
cg10141715	*SLC5A8*	12	0.06	-0.09	0.15	0.32	0.33
cg24292612	*DEFB1*	8	-0.09	0.07	-0.16	0.32	0.34
cg25538571	*FLJ46365*	8	0.03	0.15	-0.12	0.33	0.34
cg05436231	*CD164L2*	1	0.14	-0.03	0.16	0.33	0.34
cg16463460	*WT1*	11	0.06	-0.11	0.16	0.33	0.34
cg16853982	*ACTN2*	1	-0.11	0.04	-0.15	0.35	0.36
cg25345738	*PWP1*	12	-0.07	0.09	-0.15	0.35	0.36
cg14973995	*MFSD10*	4	0.04	0.16	-0.11	0.36	0.36
cg13164537	*CD226*	18	0.16	0.04	0.11	0.36	0.37
cg07260592	*LPA*	6	0.07	-0.09	0.16	0.38	0.39
cg01939428	*UBE3C*	7	-0.08	0.08	-0.16	0.41	0.41
cg07408740	*OGDH*	7	-0.07	0.08	-0.15	0.43	0.43
cg25802093	*SPAG6*	10	-0.10	0.09	-0.19	0.43	0.43
cg14603345	*BTBD3*	20	0.12	-0.05	0.17	0.43	0.44

cg13315147	*CYP2E1*	10	-0.09	-0.16	0.06	0.48	0.48

Table S3. Brain sample characteristics.

BRAIN	SOURCE	DX	SEX	AGE	PMI	REGIONS	NUCLEOTIDE	AMINO ACID
AN01570	NICHD	AUT+SEZ	F	18	7	CCTX, TCTX		
AN12457	NICHD	AUT+SEZ	F	29	18	CCTX, TCTX		
1638	NICHD	AUT+SEZ	F	20	50	CCTX, TCTX		
5278	NICHD	AUT+SEZ	F	15	13	CCTX, TCTX		
5419	NICHD	AUT+SEZ	F	19		CCTX, TCTX		
1038	NICHD	CTL	F	24	7	STRM, CCTX, TCTX		
1101	NICHD	CTL	F	19	7	CCTX, TCTX		
1614	NICHD	CTL	F	27	18	STRM, CCTX, TCTX		
1846	NICHD	CTL	F	20	9	CCTX, TCTX		
4676	NICHD	CTL	F	40	15	STRM, CCTX, TCTX		
4724	NICHD	CTL	F	16	15	STRM, CCTX, TCTX		
4725	NICHD	CTL	F	32	17	STRM, CCTX, TCTX		
5401	NICHD	CTL	F	18	22	CCTX, TCTX		
6355	HBTRC	RTT	F	16	28	STRM, CCTX, TCTX	NI	
7761	HBTRC	RTT	F	41	49	STRM, CCTX, TCTX	397C>T	R133C
7773	HBTRC	RTT	F	24	25	STRM, CCTX, TCTX	473C>T	T158M
7783	HBTRC	RTT	F	26	39	STRM, CCTX, TCTX	Exonic deletion	
B7992	HBTRC	RTT	F	31	30	STRM, CCTX, TCTX	NI	

HBTRC, Harvard Brain Tissue Resource Center; NICHD, NICHD Brain and Tissue Bank at the University of Maryland School of Medicine; PMI, post mortem interval (in hours); CCTX, cingulate cortex; STRM, striatum; TCTX, temporal cortex; NI, no mutation identified in *MECP2* coding sequence

Table S4. Reported CNVs >0.5 kb.

Sample	Chr	Start (hg18)	Stop (hg18)	Size (bp)	CNV	Reference
04C35560	9	33,669,300	33,845,500	176,200	gain	Marshall
04C35560	14	19,204,300	19,492,400	288,100	gain	Marshall
04C35560	15	18,403,700	20,089,400	1,685,700	gain	Marshall
04C36352	2	242,634,423	242,730,382	95,960	loss	Marshall
04C36352	3	60,048,193	60,263,468	215,276	loss	Marshall
04C36352	7	13,565,690	13,684,241	118,552	loss	Marshall
04C36352	14	19,272,965	19,492,423	219,459	loss	Marshall
04C36352	15	18,427,103	20,089,383	1,662,281	gain	Marshall
04C36352	17	41,518,102	41,719,833	201,732	gain	Marshall
03C15793	2	89,743,464	89,877,778	134,314	gain	Bucan
03C15793	5	45,794,921	45,866,265	71,344	gain	Bucan
03C15793	6	19,078,655	19,138,709	60,054	loss	Bucan

03C15793	6	79,029,919	79,088,461	58,542	loss	Bucan
03C15943	2	242,565,978	242,645,262	79,284	loss	Bucan
03C15943	3	75,511,364	75,613,585	102,221	loss	Bucan
03C15943	12	31,157,553	31,298,174	140,621	gain	Bucan
03C15943	15	32,505,885	32,587,887	82,002	loss	Bucan
03C15943	17	41,525,625	41,705,867	180,242	gain	Bucan
03C15943	19	47,975,959	48,387,680	411,721	loss	Bucan
03C16444	2	41,065,513	41,129,076	63,563	loss	Bucan
03C16444	6	79,029,919	79,088,461	58,542	loss	Bucan
03C16444	11	55,127,596	55,198,944	71,348	loss	Bucan
03C16444	11	67,258,201	67,505,393	247,192	loss	Bucan
03C16444	19	24,187,993	24,282,139	94,146	loss	Bucan
03C16444	19	48,337,396	48,387,680	50,284	loss	Bucan
03C16562	6	79,029,919	79,088,461	58,542	loss	Bucan
03C16562	7	110,744,529	110,980,473	235,944	loss	Bucan
03C16562	10	44,530,695	44,679,489	148,794	gain	Bucan
03C16562	11	55,127,596	55,198,944	71,348	loss	Bucan
03C16562	21	22,334,019	22,529,970	195,951	loss	Bucan
03C17196	5	97,053,241	97,121,798	68,557	loss	Bucan
03C17196	6	79,029,919	79,088,461	58,542	loss	Bucan
03C17196	11	55,127,596	55,198,944	71,348	loss	Bucan
04C24298	1	147,305,743	147,427,061	121,318	loss	Bucan
04C24298	11	55,127,596	55,198,944	71,348	loss	Bucan
04C24298	16	34,393,083	34,624,019	230,936	gain	Bucan
04C24298	19	20,422,199	20,473,895	51,696	loss	Bucan
04C24298	21	13,609,441	14,121,682	512,241	gain	Bucan
05C47170	6	79,029,919	79,088,461	58,542	loss	Bucan
05C47170	8	145,064,090	145,247,517	183,427	gain	Bucan
05C47170	11	55,127,596	55,204,003	76,407	loss	Bucan
05C47170	14	104,225,149	104,279,612	54,463	gain	Bucan
05C47170	15	32,505,885	32,587,887	82,002	loss	Bucan
05C47170	16	67,558,088	67,660,490	102,402	gain	Bucan
05C47170	16	1,366,587	1,439,378	72,791	gain	Bucan
07C64541	2	110,214,531	110,339,819	125,288	loss	Bucan
07C64541	4	82,660,183	82,725,186	65,003	gain	Bucan
07C64541	6	79,029,919	79,088,461	58,542	loss	Bucan
07C64541	X	143,125,478	143,221,467	95,989	loss	Bucan
07C64541	11	55,127,596	55,204,003	76,407	loss	Bucan
07C64541	12	31,892,781	31,954,269	61,488	gain	Bucan
07C64541	19	20,422,199	20,473,895	51,696	loss	Bucan
07C64541	22	17,257,786	17,376,565	118,779	gain	Bucan
07C64722	1	147,305,743	147,427,061	121,318	loss	Bucan
07C64722	2	241,272,566	241,332,581	60,015	gain	Bucan
07C64722	6	79,029,919	79,088,461	58,542	loss	Bucan
07C64722	11	55,127,596	55,204,003	76,407	loss	Bucan
07C64722	12	7,888,156	7,990,569	102,413	gain	Bucan
07C64722	16	1,968,402	2,028,586	60,184	gain	Bucan

Table S5. Primer sequences.

ASSAY	GENE	FORWARD 5' – 3'	REVERSE 5' – 3'
SEQ	*TAC1*	aggaagagagGATAGTGGGGAGATTGGTTTTTTA	cagtaatacgactcactatagggagaaggctCCAATAATTCAAATCATCATTAACTCC
qRT-PCR	*ACTB*	GTTGTCGACGACGAGCG	GCACAGAGCCTCGCCTT
qRT-PCR	*TAC1*	TGCACTCCTTTCATAAGCCA	AACGGGATGCTGATTCCTC
ChIP	*TAC1*	GGAGGAACCAGAGAAACTCAGC	GACCAGTAATTCAGATCATCATTGG

References

1. Bucan M, Abrahams BS, Wang K, Glessner JT, Herman EI, Sonnenblick LI, et al. (2009): Genome-wide analyses of exonic copy number variants in a family-based study point to novel autism susceptibility genes. *PLoS Genet*. 5:e1000536.
2. Marshall CR, Noor A, Vincent JB, Lionel AC, Feuk L, Skaug J, et al. (2008): Structural variation of chromosomes in autism spectrum disorder. *Am J Hum Genet.* 82:477-488.
